# Paternal MTHFR deficiency leads to hypomethylation of young retrotransposons and reproductive decline across two successive generations

**DOI:** 10.1242/dev.199492

**Published:** 2021-07-06

**Authors:** Gurbet Karahan, Donovan Chan, Kenjiro Shirane, Taylor McClatchie, Sanne Janssen, Jay M. Baltz, Matthew Lorincz, Jacquetta Trasler

**Affiliations:** 1 Department of Human Genetics, McGill University, Montreal, QC H3A 0C7, Canada; 2 Child Health and Human Development Program, Research Institute of the McGill University Health Centre, Montreal, QC H4A 3J1, Canada; 3 Department of Medical Genetics, Molecular Epigenetics Group, Life Sciences Institute, University of British Columbia, Vancouver, BC V6T 1Z3, Canada; 4 Chronic Disease Program, Ottawa Hospital Research Institute, Ottawa, ON K1H 8L6, Canada; 5 Departments of Obstetrics and Gynecology and Cellular and Molecular Medicine, University of Ottawa Faculty of Medicine, Ottawa, ON K1H 8M5, Canada; 6 Department of Pharmacology and Therapeutics, McGill University, Montreal, QC H3A 1A3, Canada; 7 Department of Pediatrics, McGill University, Montreal, QC H4A 3J1, Canada

**Keywords:** MTHFR, DNA methylation, Male germ cell development, Intergenerational epigenetic inheritance, Young retrotransposons, Mouse

## Abstract

5,10-Methylenetetrahydrofolate reductase (MTHFR) is a crucial enzyme in the folate metabolic pathway with a key role in generating methyl groups. As MTHFR deficiency impacts male fertility and sperm DNA methylation, there is the potential for epimutations to be passed to the next generation. Here, we assessed whether the impact of MTHFR deficiency on testis morphology and sperm DNA methylation is exacerbated across generations in mouse. Although MTHFR deficiency in F1 fathers has only minor effects on sperm counts and testis weights and histology, F2 generation sons show further deterioration in reproductive parameters. Extensive loss of DNA methylation is observed in both F1 and F2 sperm, with >80% of sites shared between generations, suggestive of regions consistently susceptible to MTHFR deficiency. These regions are generally methylated during late embryonic germ cell development and are enriched in young retrotransposons. As retrotransposons are resistant to reprogramming of DNA methylation in embryonic germ cells, their hypomethylated state in the sperm of F1 males could contribute to the worsening reproductive phenotype observed in F2 MTHFR-deficient males, compatible with the intergenerational passage of epimutations.

## Abstract (precis)

**Summary:** MTHFR deficiency results in sperm DNA hypomethylation of regions enriched in potentially active young retrotransposons that are targets of a piRNA- and DNMT3C-dependent *de novo* DNA methylation pathway, leading to reproductive defects that are more severe in subsequent generations.

## INTRODUCTION

DNA methylation is a well-studied epigenetic modification, generated by the addition of a methyl group to the fifth carbon of cytosine in DNA, which usually occurs in the context of CpG dinucleotides (Greenberg and Bourc'his, 2019). Male germ cells undergo widespread erasure followed by re-establishment of genomic DNA methylation patterns in the embryonic period. In the mouse, DNA demethylation takes place between embryonic day (E) 8.0 and E13.5 in primordial germ cells (PGCs), coinciding with the time when PGCs migrate and colonize the genital ridge (Kurimoto and Saitou, 2019; Sasaki and Matsui, 2008). The male germ cell genome is remethylated for the most part between E13.5 and E19.0, in mitotically arrested prospermatogonia (PSG), with some additional remodeling occurring postnatally (Kobayashi et al., 2013; Kubo et al., 2015; Molaro et al., 2014; Niles et al., 2011; Oakes et al., 2007; Seisenberger et al., 2012).

DNA methyltransferases (DNMTs), the provision of methyl groups by S-adenosylmethionine (SAM) and interactions with other epigenetic modulators, such as histone methylation, are important for the establishment of DNA methylation patterns in male germ cells. DNMT3A is the main enzyme catalyzing *de novo* DNA methylation in the male germline; it possesses an ADD domain and a PWWP domain, the latter of which binds to histone H3 methylated on H3K36 (H3K36me2/3) (Dhayalan et al., 2010; Okano et al., 1999; Weinberg et al., 2019). The PWWP domain of DNMT3A is postulated to mediate the crosstalk between H3K36 methylation and DNA methylation. Indeed, mutations in the PWWP domain of DNMT3A disrupt the interaction between DNMT3A and H3K36me2, and lead to aberrant targeting of DNMT3A (Weinberg et al., 2019). DNMT3A-dependent *de novo* DNA methylation in prenatal male germ cells is also dependent upon the catalytically inactive paralog DNMT3L (Zhang et al., 2018; Chédin et al., 2002). Although DNMT3L lacks the PWWP domain, it does contain an ADD domain, which binds to unmethylated H3K4. DNMT3C, a recently discovered rodent-specific member of the DNMT3 family, is essential for *de novo* DNA methylation and silencing of young retrotransposons during spermatogenesis (Barau et al., 2016).

Although the roles of the different DNMTs in male germ cell DNA methylation have been studied in detail, less is known regarding the effects of altering methyl group availability. 5,10-Methylenetetrahydrofolate reductase (MTHFR), an enzyme involved in the production of a major source of methyl groups, SAM, has received attention in the context of male germ cell DNA methylation owing to its connection to male infertility. MTHFR reduces 5,10-methylenetetrahydrofolate (5,10-methyleneTHF) to 5-methylTHF, the primary carbon donor for methionine production from homocysteine. Notably, MTHFR is highly expressed in mouse testes starting at E15 (Garner et al., 2013) compared with other adult tissues (Chen et al., 2001). A common genetic variant in humans (*MTHFR* 677C >T) results in a thermolabile enzyme, with ∼50% reduced enzymatic activity (Kang et al., 1991). The *MTHFR-677TT* genotype has been associated with idiopathic male infertility in some populations (Bezold et al., 2001; Hong et al., 2017).

Mouse studies have revealed a crucial role for MTHFR in male germ cells. MTHFR is most highly expressed in male germ cells at the time of DNA methylation acquisition in the embryonic gonad (Garner et al., 2013). The effect of MTHFR deficiency on fertility in mice has been studied using mice with a targeted mutation (null allele) in the *Mthfr* gene. *Mthfr*^−/−^ mice on a C57BL/6 background were healthy and fertile, but had reproductive abnormalities, including lower testis weights, lower sperm counts and increased proportions of abnormal seminiferous tubules (Chan et al., 2010; Lawrance et al., 2011). In contrast, *Mthfr*^−/−^ mice on a BALB/c background showed more severe reproductive defects and were infertile, indicating an important role of genetic background, as suggested by human studies. Analysis of sperm of C57BL/6 *Mthfr*^−/−^ mice using a low-resolution DNA methylation analysis technique, revealed preliminary evidence of effects of MTHFR deficiency on sperm DNA methylation (Chan et al., 2010).

**Fig. 1.**
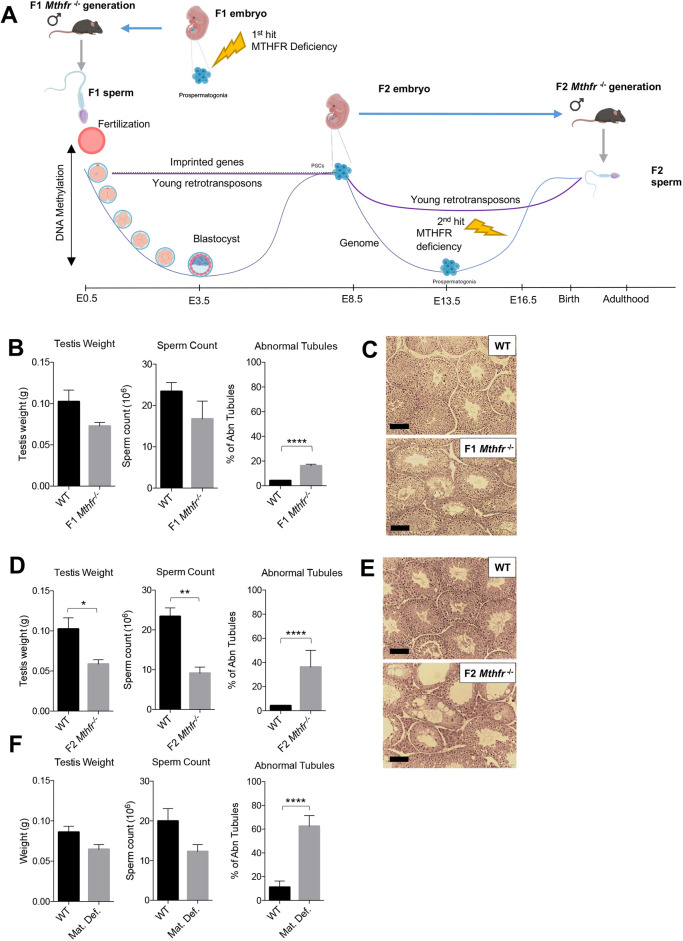
**MTHFR deficiency impacts epigenetic reprogramming in male germ cells and results in reproductive decline across two generations.** (A) Experimental design for the production of F1 and F2 MTHFR-deficient males. Based on high levels of expression of MTHFR in prospermatogonia (PSG), MTHFR deficiency is expected to affect F1 generation (1st hit) primordial germ cells (PGCs) when DNA methylation patterns are established. Epimutations in the sperm of MTHFR-deficient fathers will either be corrected postfertilization during reprogramming in F2 pre-implantation embryos or, if they escape reprogramming, be passed on to the germ cells of the F2 post-implantation MTHFR-deficient embryos (sons). A second phase of reprogramming takes place in the PGCs of the F2 MTHFR-deficient sons, preceding re-acquisition of DNA methylation in PSG, which are deficient in MTHFR (2nd hit). If PGC reprogramming is not complete, F2 generation PSG may carry epimutations from their fathers in addition to being impacted by MTHFR at the time of *de novo* methylation. (B) Testis weight, sperm count and proportion of abnormal testicular tubules in *Mthfr*^+/+^ (WT) and *Mthfr^−/−^* F1 males (WT, *n*=3; F1 *Mthfr^−/−^*, *n*=4). (C) Representative histological cross-sections of testes. (D) Testis weight, sperm count and proportion of abnormal testicular tubules in F2 generation males (WT, *n*=3; F2 *Mthfr^−/−^*, *n*=4). (E) Representative testicular histological cross-sections from F2 generation males. (F) Testis weight, sperm count and proportion of abnormal testicular tubules in the maternal deficient (Mat. Def.) group of WT (*n*=4) and *Mthfr^−/−^* (*n*=6) males. Data are mean+s.e.m. *P<0.05; **P<0.01; ****P<0.0001. Scale bars: 100 µm.

Together, human and mouse studies have suggested an important role for MTHFR in normal male fertility as well as the coincidence of its expression with DNA methylation reprogramming in embryonic male germ cell development. However, effects of MTHFR deficiency on DNA methylation in sperm and the consequences for the next generation have not been studied. Using MTHFR-deficient C57BL/6 male mice (fathers) and their second generation MTHFR-deficient offspring (sons), we examined reproductive health and employed a sensitive genome-wide approach to investigate the effects of MTHFR deficiency on sperm DNA methylation across two generations (Fig. 1). In addition, a maternal MTHFR-deficient cohort was used to determine whether the methylation changes observed in the next generation were specific to paternal MTHFR deficiency and rule out the potential confounding effect of methyl donors supplied by mothers with normal MTHFR activity. We hypothesized that if DNA methylation abnormalities are reprogrammed between generations, sons would show effects similar to those observed in their fathers. In contrast, if epimutations accumulate across generations, sons would be expected to be more affected than their fathers. Here, we show that second generation males show more evidence of reproductive defects than males of the first generation and identify DNA methylation defects in sperm that provide a potential explanation for the findings.

## RESULTS

### General health and reproductive parameters across successive generations in MTHFR-deficient male mice

To determine the effects of MTHFR deficiency across two generations, F0 founder *Mthfr^+/−^* males and females were bred to obtain F1 generation *Mthfr^−/−^* and *Mthfr^+/+^* wild-type (WT) male mice. Next, F1 generation *Mthfr^−/−^* (fathers) and WT males were mated with *Mthfr^+/−^* females to acquire F2 generation *Mthfr^−/−^* and WT male mice (sons). As illustrated in Fig. 1A, the F2 MTHFR-deficient sons received two potential hits to their testes and sperm DNA methylation: one due to their fathers' MTHFR deficiency during germ cell development and the second due to the absence of MTHFR in their own germ cells (Fig. 1A). First, we assessed the F1 generation male mice. Body weight of adult mice was measured as an indicator of their general health. This revealed a similar weight between *Mthfr^−/−^* and WT animals in both generations, indicating that MTHFR loss does not affect the general health of male mice (Fig. S1A). In addition, we determined the effects of MTHFR deficiency on the testis, the tissue with the highest level of MTHFR expression (Chen et al., 2001). Consistent with our previous report (Chan et al., 2010), a decrease in testis weight of approximately 30% was seen in F1 *Mthfr^−/−^* males compared with WT males. Similarly, testicular sperm counts were reduced (Fig. 1B). However, the decreases in weight and sperm count were not statistically significant. Examination of testicular histology revealed a significant increase of ∼20% in the proportion of morphologically abnormal seminiferous tubules in testes of MTHFR-deficient F1 mice (Fig. 1B,C). Thus, lifetime MTHFR deficiency leads to testicular abnormalities without affecting the overall health of the males, such that they are able to produce the F2 generation.

Next, we assessed the reproductive health of the F2 generation *Mthfr^−/−^* males and their WT counterparts. F2 *Mthfr^−/−^* adult males were healthy with body weights similar to WT males (Fig. S1A). Because the DNA methylome of the F2 MTHFR-deficient sons undergoes two waves of demethylation and remethylation, we anticipated that reproductive parameters in the sons would resemble those of their fathers. Surprisingly, testis weights and sperm counts were more severely affected in the F2 MTHFR-deficient sons compared with their fathers (Fig. 1D). In line with these results, histological examination of the testes of F2 MTHFR-deficient sons revealed a higher number of abnormal seminiferous tubules compared with their fathers (Fig. 1D,E). Of note, F2 generation males mated with WT females for a period of 3 months were unable to produce litters, suggesting that the F2 males are subfertile or infertile. Together, these results indicate that the testes of F2 generation MTHFR-deficient sons are more severely affected than the testes of their *Mthfr^−/−^* fathers.

The father and son experiments depicted in Fig. 1A and described above made use of *Mthfr*^+/−^ females to obtain the F1 and F2 males. In such pregnancies, one-carbon metabolism in the mothers of MTHFR-deficient and WT males would potentially maintain normal SAM levels, which could in turn be transferred to fetuses and metabolized by developing germ cells. Therefore, we investigated whether DNA methylation in the sperm of F2 males (i.e. those with *Mthfr*^−/−^ fathers) could be further impacted by maternal MTHFR deficiency. F2 males were derived from matings between *Mthfr*^−/−^ males and females (Fig. S2A). Consistent with the findings in the F2 sons reported above, the resulting maternal-deficient (Mat. Def.) F2 *Mthfr*^−/−^ males showed a 25% decrease in testis weights, a 40% decrease in testicular sperm counts, and >50% increase in the proportion of abnormal tubules compared with WT males (Fig. 1F). The WT males showed similar reproductive parameters (testis weights, sperm counts, testis histology) to those of WT males in the father/son experiments. Thus, reproductive parameters in these Mat. Def. F2 generation males are minimally impacted by maternal MTHFR deficiency.

### F1 generation *Mthfr^−/−^* fathers and F2 generation *Mthfr^−/−^* sons show a profound loss of sperm DNA methylation

Given the crucial role of MTHFR in SAM production, as well as the high level of MTHFR expression during key periods of DNA methylation acquisition and maintenance, MTHFR likely impacts DNA methylation patterns in sperm. To examine the effect of MTHFR loss on sperm DNA methylation in F1 as well as F2 *Mthfr^−/−^* males, we used reduced representation bisulfite sequencing (RRBS).

**Fig. 2.**
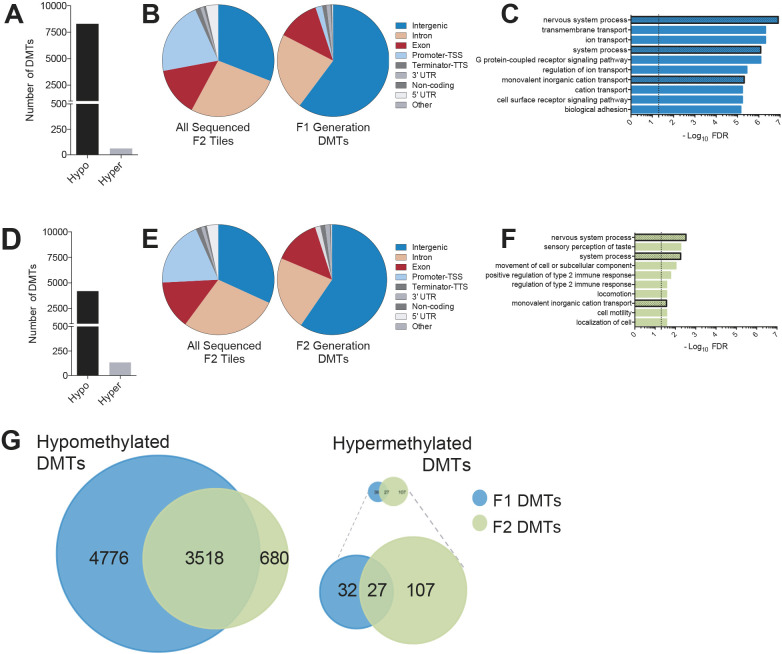
**Genome-wide loss of sperm DNA methylation in MTHFR-deficient F1 and F2 generation males.** (A) Number of 100 bp tiles that significantly lost (hypomethylated) or gained (hypermethylated) methylation in the sperm of F1 *Mthfr^−/−^* compared with WT males. (B) Distribution of DMTs into genomic elements is shown for all sequenced F1 tiles as well as F1 generation DMTs. (C) GO enrichment analysis of genic DMTs in F1 generation males. The dotted line indicates the *P*<0.05 threshold for significance for FDR. The dotted bars indicate common enriched pathways between the F1 and F2 generations. (D) Number of 100 bp tiles that were significantly hypomethylated or hypermethylated in the sperm of F2 *Mthfr^−/−^* compared with WT males. (E) Distribution of DMTs into genomic elements is shown for all sequenced F2 tiles as well as F2 generation DMTs. (F) GO enrichment analysis of genic DMTs in F2 generation males. The dotted line indicates the *P*<0.05 threshold for significance for FDR. The shaded bars indicate common enriched pathways between the F1 and F2 generations. (G) Euler diagrams of common hypo- and hypermethylated tiles between sperm of F1 and F2 generation males. Hypermethylated tiles are shown proportional (in size) to the hypomethylated tiles on the top right, with the magnified version shown below.

RRBS revealed thousands of differentially methylated tiles (DMTs, 100 bp/tile) for F1 generation *Mthfr^−/−^* compared with WT males (8359 tiles). The vast majority of these tiles showed a loss of methylation (hypomethylation; 8296, 99.2%) and only few tiles demonstrated a gain in methylation (hypermethylation; 63, <1%) (Fig. 2A, Table S1). DMTs were mainly located in intergenic (60.1%), intronic (22.3%) and exonic (12.3%) regions. The remaining genomic locations (promoter, terminator-TSS, 3′UTR, 5′UTR, non-coding, others) contained only 5.3% of the DMTs (Fig. 2B). The most notable difference between the genomic distribution of all sequenced tiles and that of F1 DMTs was the two-fold over-representation of DMTs in intergenic regions (Fig. 2B), suggesting that intergenic DNA methylation in sperm is most susceptible to the loss of MTHFR.

To assess further the impact of MTHFR deficiency on sperm DNA methylation, we examined the magnitude of change for the identified DMTs. Although most DMTs showed a magnitude of methylation change in the range of 10-20% (69.6%), a significant number (30.4%) of DMTs showed changes >20% in magnitude (Fig. S1B). Using genic DMTs (excluding the intergenic regions), we performed gene ontology (GO) analysis to identify over-represented biological processes, employing the web-based functional enrichment analysis tool WebGestalt (WEB-based GEne SeT AnaLysis Toolkit) (Liao et al., 2019). This revealed a large number of GO biological processes as significantly over-represented based on DMTs in sperm of the F1 generation MTHFR-deficient males. The top GO term was ‘nervous system process’ with many others related to ion transport pathways (Fig. 2C).

As F2 generation *Mthfr^−/−^* males derived from F1 males with hypomethylated sperm DNA (first hit) lack MTHFR in their germ cells (second hit, Fig. 1A), we expected the sperm DNA methylome of the F2 sons to be similarly or more severely affected than that of their fathers. Indeed, consistent with the MTHFR-deficient F1 fathers, the sperm of F2 sons was predominantly hypomethylated (96.7% of DMTs) compared with WT (Fig. 2D). However, unexpectedly, we observed ∼50% fewer DMTs (4332) in F2 versus F1 generation sperm (Table S1). The distribution of DMTs in the genome of F2 sperm resembled that found in F1 sperm, with alterations in DNA methylation affecting mostly intergenic regions (59.6%), followed by intronic (21.7%) and exonic (13.7%) regions (Fig. 2E). Similar to our findings in F1 *Mthfr*^−/−^ males, intergenic regions were approximately two-fold enriched in F2 sperm compared with all F2 sequenced tiles (Fig. 2E), further highlighting the potential role for MTHFR in DNA methylation at intergenic regions.

Analysis of the magnitude of DNA methylation changes for F2 generation DMTs revealed significantly more DMTs in the range of 10-15% (56.1%) compared with F1 generation DMTs. Surprisingly, significantly fewer F2 DMTs showed DNA methylation changes >20% in magnitude (23.5%) compared with F1 generation DMTs in this range (Fig. S1C). Next, genic F2 DMTs were used to identify enriched pathways and, interestingly, three of the top ten pathways (‘nervous system process’, ‘system process’ and ‘monovalent inorganic cation transport’) were shared between F1 and F2 generations (Fig. 2C,F).

To explore further the shared effects of MTHFR deficiency in the F1 and F2 sperm DNA methylomes, we determined whether specific tiles are shared between both generations. For hypermethylated DMTs, this was only the case for a small subset of DMTs (Fig. 2G, right). However, strikingly, 83.8% of all hypomethylated DMTs in F2 sperm were also found in F1 sperm (Fig. 2G, left), suggesting that these regions are consistently susceptible to MTHFR deficiency.

Next, we used RRBS to determine DNA methylation changes in the Mat. Def. F2 MTHFR-deficient males and compared them with the DMTs of F1 fathers and F2 sons as described above. Sperm of the Mat. Def. F2 MTHFR-deficient males showed changes in 2709 DMTs with the majority (95% of DMTs) showing hypomethylation (Fig. S2B, Table S1). The genomic distribution of DMTs was similar to that found in the F1 and F2 father/son experiments (Fig. S2C). However, the magnitude of changes was significantly different compared with F1 (fathers) in all categories (10-15%, 15-20%, 20-25%, 25-30%, 30-40% and >40%) and F2 (sons) in all but the 20-25% category (Fig. S2D). Genic DMTs were used to identify enriched biological processes. Similar to the F1/F2 father/son experiment, ‘nervous system process’ and ‘system process’ were also in the top 3 enriched pathways (Fig. S2E). To understand better the extent to which maternal MTHFR deficiency contributes to sperm DNA methylation changes compared with the F1 and F2 generations, we intersected the DMTs in all three populations. This analysis revealed 892 common DMTs in all three populations (878 hypo- and 14 hypermethylated), constituting 25.1% of 3545 common DMTs between the F1 and F2 father/son generations (Fig. S2F). These data suggest that effects of MTHFR deficiency on sperm DNA methylation are most similar in the F2 sons and the Mat. Def. F2 males (in each case, mice resulting from two generations of MTHFR deficiency).

### MTHFR deficiency alters sperm DNA methylation at a regional level

**Fig. 3.**
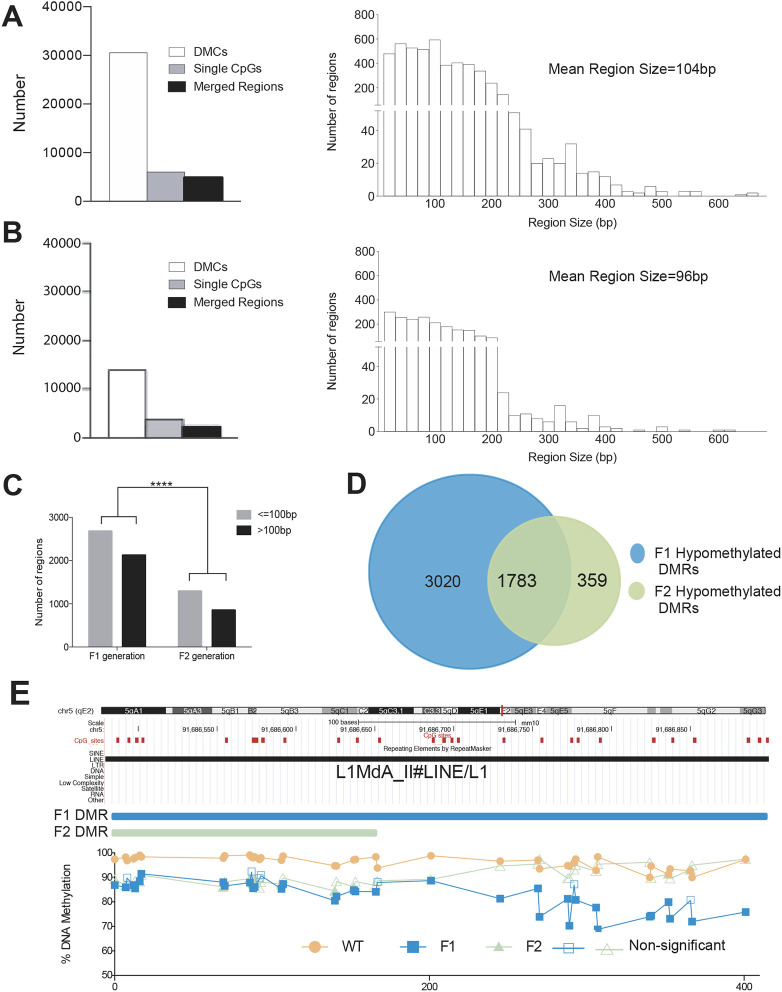
**MTHFR-associated sperm hypomethylation extends beyond isolated CpGs to encompass larger regions in F1 and F2 generation males.** (A,B) Comparison of sperm hypomethylated DMRs in MTHFR-deficient males of the F1 (A) and F2 (B) generations. Left: The number of all differentially methylated CpG sites (DMCs), single isolated CpGs (not merged into regions) and merged regions acquired by adjoining DMCs within 100 bp from each other. Right: The distribution of merged DMRs. (C) Comparison of sizes of hypomethylated merged regions (regions equal to or smaller than 100 bp and larger than 100 bp) between F1 and F2 generations. ****P<0.0001. (D) Euler diagram of the common sperm hypomethylated merged DMRs between the F1 and F2 generations. (E) A large sperm hypomethylated DMR in MTHFR-deficient males showing an example of an F2 region within an F1 region (a L1MdA repeat region) is shown on chromosome 5, with CpG sites in the region indicated as red boxes. In the graph, filled shapes indicate significant DMCs and unfilled shapes indicate non-significant DMCs.

Given the profound changes in DNA methylation observed above, we set out to examine the effect of MTHFR loss on larger-scale regional changes in DNA methylation, beyond those of 100 bp tiles. To achieve this, we merged neighboring differentially methylated CpGs (DMCs), located within 100 bp of each other, into regions. We focused our analysis on hypomethylated DMCs, as the majority of DMTs described above were hypomethylated (Fig. 2A,D). Using this approach, only 19.6% of all F1 hypomethylated DMCs were devoid of any neighboring DMCs and identified as single CpGs. The remainder of DMCs (24,559) were merged into 4803 differentially methylated regions (DMRs) (Fig. 3A, left). For the F1 generation fathers, distribution of DMRs according to their size showed that most of the regions were in the 10-200 bp range. However, a number of larger regions were seen, with the largest being 664 bp in size (Fig. 3A, right). Compared with the F1 fathers, the F2 generation MTHFR-deficient sons showed fewer DMCs (13,879), which is consistent with the lower number of DMTs detected in the F2 *Mthfr^−/−^* sons. Of these DMCs, 27.1% were found as single CpGs. The remaining CpGs could be assembled into 2142 regions, revealing that most of these regions are smaller than 200 bp in size (Fig. 3B, right), which is similar to the F1 *Mthfr*^−/−^ fathers. In addition, some larger regions were observed, the largest one 614 bp in size (Fig. 3B, right).

Next, we assessed whether there is a difference in the distribution of DMRs that are ≤100 bp and >100 bp between F1 and F2 *Mthfr*^−/−^generations. The number of DMRs greater than 100 bp was significantly higher for F1 sperm compared with F2 sperm (Fig. 3C). Similar to the shared hypomethylated DMTs in the F1 and F2 *Mthfr*^−/−^ generations (83.8%, Fig. 2G), we found that 83.2% of F2 hypomethylated DMRs overlap with F1 generation DMRs (Fig. 3D). Of these shared DMRs, 38.5% showed a 100% overlap, 46.6% were F2 DMRs located within F1 DMRs, 9.6% represented F1 DMRs within F2 DMRs and 5.4% showed a partial overlap (Fig. S3A). An example of the most common type of overlap, an F2 DMR within an F1 DMR, is shown in Fig. 3E for a young retrotransposon belonging to the L1MdA_II family of long interspersed nuclear element-1s (LINE-1).

In addition to the F1 father and F2 sons, regions were merged for the Mat. Def. F2 MTHFR-deficient group, resulting in 919 DMRs averaging 86 bp in size (Fig. S3B). Out of the 919 hypomethylated DMRs, 360 (39%) overlapped with the DMRs observed in F1/F2 father/son sperm (Fig. S3C). These data suggest that maternal MTHFR loss in addition to paternal MTHFR loss does not lead to accumulative DNA methylation changes in the F2 sons.

Thus, although similar regions in the genome lose DNA methylation in sperm from F1 and F2 generation *Mthfr*^−/−^ male mice, the magnitude of DNA methylation loss is lower in the F2 sons compared with the F1 fathers. In addition, there are remarkable overlaps in regions affected in all three groups of males.

### MTHFR-sensitive DMR regions are predominantly located in intergenic, open sea regions, and are enriched in young retrotransposons

**Fig. 4.**
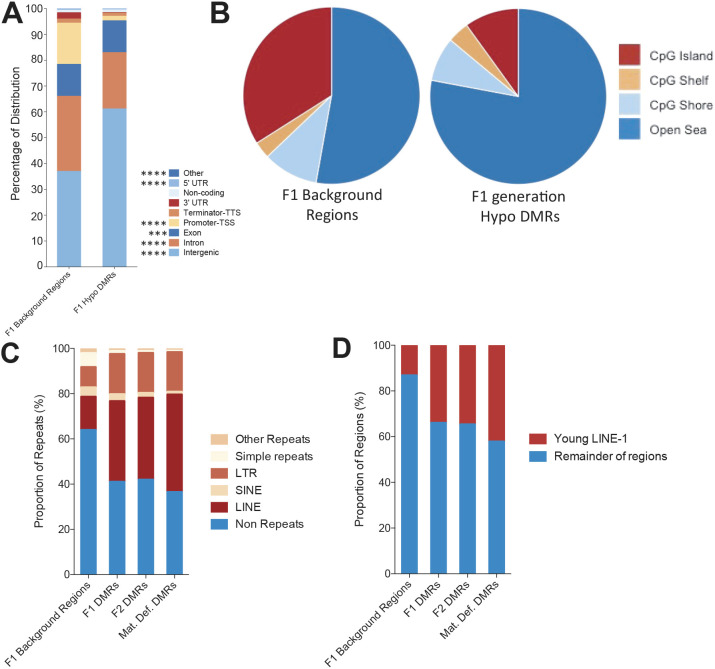
**MTHFR-sensitive sites are enriched for intergenic sequences and young retrotransposons.** (A) Distribution of F1 merged hypomethylated DMRs into genomic elements in comparison with all sequenced F1 regions. ***P<0.001; ****P<0.0001. (B) Location of F1 merged hypomethylated DMRs with respect to CpG islands/shores/shelves and open sea regions in comparison with all sequenced F1 regions. (C) The proportion of overlaps between merged hypomethylated DMRs with regions identified as repeats using the RepeatMasker program (LINE, long interspersed nuclear element; LTR, long terminal repeat; SINE, short interspersed nuclear element). (D) The proportion of overlaps between merged hypomethylated DMRs with young LINEs (L1Md family of retrotransposons, Fig. S5) compared with the remainder of the regions.

Extensive overlaps of MTHFR-sensitive sperm hypomethylated regions between the F1 and F2 generations and the Mat. Def. group (Fig. S3C) led us to examine the specific sequences in these regions more closely. The distribution of hypomethylated DMRs in all groups was largely enriched for intergenic regions compared with their respective all sequenced merged regions (Fig. 4A, Fig. S3D). In accordance with this enrichment in intergenic regions, DMRs occupied open sea regions with low CpG density, located >4 kb away from any CpG islands (Fig. 4B, Fig. S3E). Amongst the DMRs, we observed a very significant enrichment of LINE-1 and short interspersed nuclear elements (SINE) in the sperm from all groups of males with MTHFR deficiency compared with F1 all sequenced background regions (Fig. 4C; F2 and Mat. Def. all sequenced background regions are almost identical to F1 and are omitted from Fig. 4C,D for simplicity). We hypothesized that the observed reproductive deterioration in MTHFR-deficient animals across generations might be due to a stochastic loss of DNA methylation and transcriptional activation of young LINE-1 elements, subsequently causing germ cell death. Our DMRs were further examined for enrichment of the L1Md subfamily of LINE-1 elements, which contains most young families of retrotransposon-competent L1 elements (Fig. S5). DMRs in all three groups were significantly enriched in these young retrotransposons (Fig. 4D).

### MTHFR-sensitive DMRs coincide with genomic regions subject to late DNA methylation in prospermatogonia and marked by H3K4me3

**Fig. 5.**
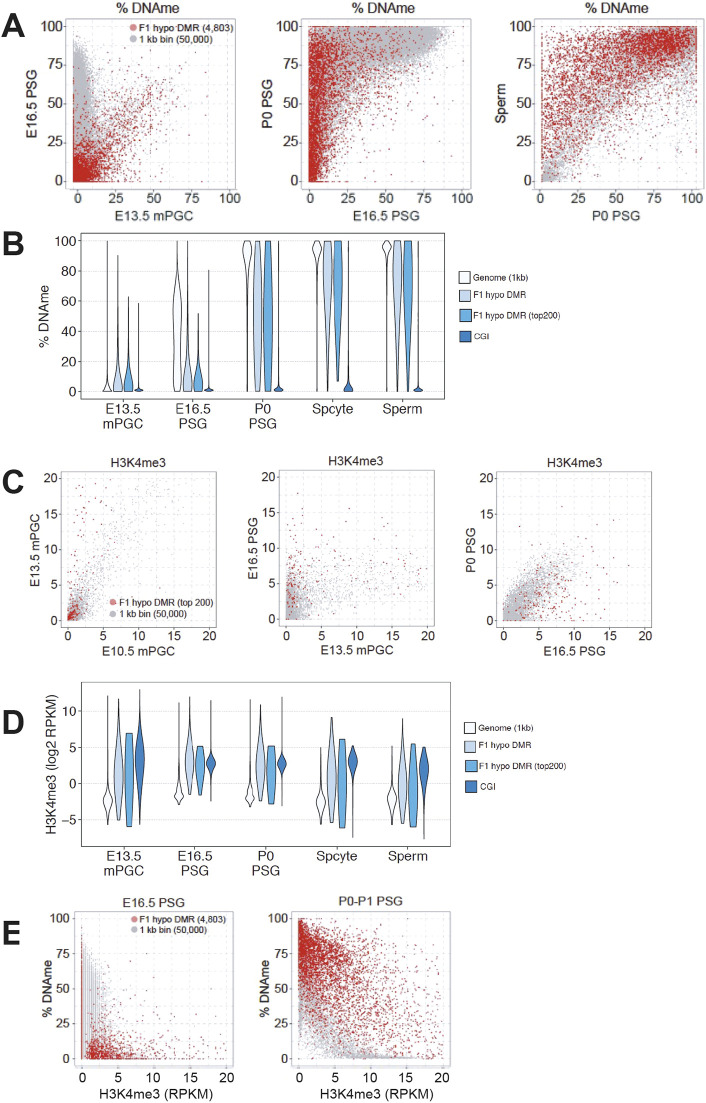
**MTHFR-sensitive sites are subject to late *de novo* methylation and marked by H3K4me3.** (A) Scatterplots showing percentage DNA methylation (DNAme) in 4803 F1 hypomethylated DMRs (red dots) compared with whole-genome 1 kb bins (gray dots) in E13.5 male PGCs versus E16.5 PSG (left), E16.5 PSGs versus P0 PSG (middle) and P0 PSG versus sperm (right). For genome 1 kb bins, 50,000 randomly selected data points are plotted. (B) Violin plots showing the distribution of the percentage DNAme levels of whole-genome 1 kb bins, F1 hypo DMRs, top 200 F1 hypo DMRs (by size) and CpG islands (CGIs) during spermatogenesis, including spermatocytes (Spcyte). (C) Scatterplots of H3K4me3 levels in the top 200 F1 hypomethylated DMRs (red dots) compared with whole-genome 1 kb bins (gray dots) in E10.5 versus E13.5 PGCs (left), E13.5 PGCs versus E16.5 PSGs (middle) and E16.5 versus P0 PSGs (right). (D) Violin plots showing the distribution of H3K4me levels for whole-genome 1 kb bins, F1 hypo DMRs, the top 200 F1 hypo DMRs (by size) and CGIs during spermatogenesis, including spermatocytes and sperm. (E) Scatterplots showing the percentage of DNAme versus H3K4me3 levels at F1 hypomethylated DMRs (red dots) compared with whole-genome 1 kb bins (gray dots) for E16.5 PSG (left) and P0-P1 PSG (right). RPKM, reads per kilobase million.

During DNA methylation acquisition in male germ cells, a subset of genomic sequences is methylated early, whereas others are methylated later in development. Our previous study indicated that methylation of the paternally methylated imprinting control regions (ICRs) of the imprinted genes *H19* and *Dlk1-Gtl2* (*Meg3*), sequences known to acquire methylation early during the wave of DNA methylation in male germ cells, are not affected by MTHFR deficiency (Chan et al., 2010). We postulated that sequences that are hypomethylated in MTHFR-deficient germ cells might be subject to different methylation dynamics than that of imprinted ICRs. We used publicly available whole-genome bisulfite sequencing (WGBS) data on isolated germ cells from different developmental time points [E13.5, E16.5, postnatal day (P) 0 and sperm] to determine the normal timing of methylation of the MTHFR-sensitive sequences (Kobayashi et al., 2013; Kubo et al., 2015; Shirane et al., 2020). Substantial increases in DNA methylation occurred across the genome between E13.5 and E16.5, with nearly complete acquisition by P0 (Fig. 5A,B). In contrast, sequences subject to hypomethylation in the F1 MTHFR-deficient males (*n*=4803) showed a dramatic gain in methylation only after E16.5, with further increases continuing in germ cells developing from P0 spermatogonia through to mature sperm (Fig. 5A,B). This pattern of late methylation was also found for sequences hypomethylated in F2 generation MTHFR-deficient sperm (*n*=2142) as well as sequences commonly affected in both the F1 and F2 generations (Fig. S4A-C).

Next, we investigated whether specific histone modifications might be enriched at the MTHFR-sensitive sites showing such late DNA methylation. As H3K4me3 is known to block DNA methylation due to its binding preference of the ADD domain of DNMT3L towards unmethylated H3K4 (Zhang et al., 2010), the presence of this histone mark is a plausible candidate for explaining the delayed acquisition of DNA methylation marks at MTHFR-sensitive sequences. We used published chromatin immunoprecipitation (ChIP) sequencing data on isolated E10.5, E13.5 and E16.5 male germ cells to compare H3K4me3 enrichment at a genome-wide level versus that for sequences affected by MTHFR deficiency (Kawabata et al., 2019; Shirane et al., 2020). Compared with the whole genome, where H3K4me3 was relatively stable, MTHFR-sensitive sequences showed increased levels of H3K4me3 in 1-kb bins from E13.5 to E16.5 (Fig. 5C,D). CpG-islands (CGI), which normally remain unmethylated throughout spermatogenesis, were also enriched in H3K4me3. Comparing DNA methylation levels with H3K4me3 marks at E16.5 and P0 clearly showed that higher levels of retention of H3K4me3 coincide with sites of late DNA methylation for MTHFR-sensitive genomic regions (Fig. 5E, Fig. S6A-C). Together, these results suggest that H3K4me3 methylation may protect MTHFR-sensitive sites from acquiring DNA methylation by E16.5.

### MTHFR-sensitive sites are preferential targets of the DNMT3C-dependent *de novo* DNA methylation pathway

The absence of MTHFR in mitotically arrested prospermatogonia in *Mthfr^−/−^* males could potentially limit the availability of methyl donors for histone or DNA methylation reactions, in turn leading to DNA hypomethylation. In a recent study, we proposed that there are two DNMT3L-dependent pathways underlying *de novo* methylation in male germ cells, one that is guided by H3K36me2/3 and a second that is limited to evolutionarily young transposable elements (TEs), guided by piRNAs and requiring DNMT3C (Shirane et al., 2020). This study showed that whereas SETD2, the enzyme that deposits H3K36me3, is dispensable for *de novo* methylation in prospermatogonia, the related H3K36 dimethyltransferase NSD1, which deposits H3K36me2, plays an essential role in the acquisition of DNA methylation in prenatal male germ cells. However, *de novo* methylation of young TEs marked with H3K4me3 in E16.5 prospermatogonia is relatively unaffected in *Nsd1* knockout (KO) mice, likely explained by the fact that young TEs are targeted for DNA methylation by DNMT3C (Barau et al., 2016).

**Fig. 6.**
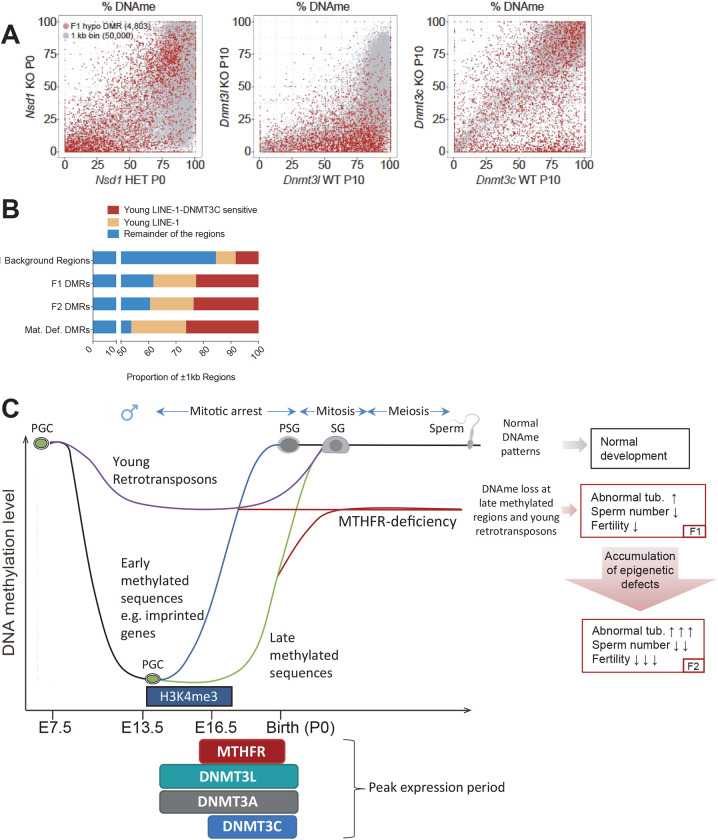
**MTHFR-sensitive sites are targets for DNMT3l and DNMT3C.** (A) Scatterplots showing the percentage DNA methylation in 4803 F1 hypomethylated DMRs (red dots) compared with 50,000 whole-genome 1 kb bins (gray) in *Nsd1* heterozygotes (HET) versus knockout (KO) P0 PSG (left) (Shirane et al., 2020), *Dnmt3l* wild-type (WT) versus KO P10 spermatogonia (middle) and *Dnmt3c* WT versus KO P10 spermatogonia (right) (Barau et al., 2016). (B) The proportion of DMRs (±1 kb) overlapping with DNMT3C-sensitive regions that showed more than 5-fold increased expression in *Dnmt3c* KO compared with *Dnmt3c*^+/−^ (DNMT3C sensitive) (Barau et al., 2016) in P20 testes, within young LINEs compared with the remainder of the regions. (C) Proposed model showing the effect of MTHFR deficiency in male germ cells during development. *De novo* DNA methylation patterns are established normally in male germ cells when MTHFR is expressed at normal high levels in prospermatogonia (blue line). MTHFR is normally expressed at high levels during the phase of late *de novo* DNA methylation and thus deficiency of MTHFR is predicted to limit methyl donor levels and preferentially impact H3K4me3-marked sequences that would normally be methylated by DNMT3L and DNMT3C after E16.5 days. The worsening of reproductive parameters in MTHFR-deficient sons versus their fathers suggests that epigenetic defects can accumulate across generations. The preferential loss of DNA methylation at young retrotransposons, sequences that are normally kept highly methylated through both PGC and pre-implantation reprogramming phases, could contribute to this effect. Loss of DNA methylation at these specific regions could potentially result in an increased expression of young retrotransposons and lead to germ cell death and subfertility. tub., tubules.

Published WGBS data from germ cells of males with NSD1, DNMT3L or DNMT3C deficiency were examined to identify the enzymes involved in *de novo* methylation at MTHFR-sensitive sites (Fig. 6A). Although DNA methylation at 50,000 randomly selected 1 kb regions was broadly reduced in *Nsd1* KO compared with *Nsd1* heterozygous prospermatogonia, there was little effect of the *Nsd1* KO on the 4803 MTHFR-sensitive F1 sites (Fig. 6A, left). These results indicate that NSD1-mediated H3K36me2 is not the major driver of the *de novo* methylation at MTHFR-sensitive sites. In contrast, a similar analysis of germ cells from *Dnmt3l* KO mice (Fig. 6A, middle) revealed decreased DNA methylation at both randomly selected 1 kb regions as well as the vast majority of MTHFR-sensitive sites, consistent with a previous report showing that *de novo* DNA methylation in the male germline is broadly dependent on DNMT3L (Barau et al., 2016). Analysis of germ cells from *Dnmt3c* KO mice, by contrast, revealed minimal impact on DNA methylation levels across the genome, but a clear decrease in DNA methylation at a subset of MTHFR-sensitive sites (Fig. 6A, right). Similar results were obtained for the top 200 F1 MTHFR-sensitive sites, the F2 MTHFR hypomethylated sites and the F1-F2 common sites (Fig. S7A-C). Together, these results indicate that DNA methylation at MTHFR-sensitive sites depends upon DNMT3L, whereas DNMT3C is required for DNA methylation of only a subset of these sites.

Given that young TEs are specifically targeted for DNA methylation by DNMT3C (Barau et al., 2016), we next mined RNA-seq data from *Dnmt3c* KO and WT spermatogonia to determine which TEs were at least 5-fold upregulated upon DNMT3C loss. As our DMRs were highly enriched in LINE-1 elements, we focused on them. We identified eight subfamilies (L1MdA_I, L1MdTf_I, L1MdTf_II, L1MdA_II, L1MdGf_I, L1MdTf_III, L1MdGf_II and L1MdA_III) as DNMT3C-sensitive LINE-1 elements (Fig. S5) (Barau et al., 2016). MTHFR-sensitive DMR regions (within ±1 kb of DMR) were used to identify overlaps with the DNMT3C-sensitive LINE-1 elements. We found that for all three MTHFR-deficient groups, there was enrichment for young LINE-1 elements, in particular those that were sensitive to *Dnmt3c* deletion (Fig. 6B). These results indicate that MTHFR-sensitive sites are highly enriched in young LINE-1 elements, potentially regulated by the DNMT3C/piRNA pathway, which acts relatively late in embryonic male germ cell development.

## DISCUSSION

In this study, genome-wide DNA methylation analysis reveals DNA hypomethylation of sperm DNA in both F1 and F2 MTHFR-deficient mice. Importantly, we observed an extensive overlap between hypomethylated DMRs in the two generations. These hypomethylated DMRs correspond to genomic regions that, during DNA methylation reprogramming in the male germline, acquire *de novo* DNA methylation at a later time point compared with the rest of the genome. Of note, the *de novo* DNA methylation of these regions coincides with the expression of MTHFR in prenatal male germ cells. Most strikingly, MTHFR-sensitive regions were mainly found on or in close proximity to evolutionarily young retrotransposons in the genome, elements that are known to escape reprogramming in germ cells and pre-implantation embryos. Based on the identity of DNA hypomethylated sites, we propose a model that may help explain why testicular defects in F1 MTHFR-deficient mice are amplified in F2 sons.

In line with our previous study, MTHFR deficiency results in testicular abnormalities in the first generation (Chan et al., 2010). We initially hypothesized that any epigenetic defects found in the sperm of MTHFR-deficient F1 males would be erased as a result of the epigenetic reprogramming that occurs during pre-implantation development, resulting in F2 sons with a testicular phenotype similar to their fathers. However, unexpectedly, the reproductive deterioration in the F2 sons instead suggests that epimutations from the fathers are resistant to epigenetic reprogramming.

MTHFR is first expressed in prospermatogonia at E15.0 (Garner et al., 2013), coinciding with germ-cell specific *de novo* DNA methylation. In our previous study, we showed not only reproductive defects in MTHFR-deficient C57BL/6 male mice but also evidence of altered sperm DNA methylation using a low-resolution DNA methylation analysis technique (Chan et al., 2010). Hence, here we focused on DNA methylation as a possible transmission mechanism of epigenetic defects from F1 fathers to their sons. We used a higher resolution, genome-wide DNA methylation analysis technique to evaluate better the extent of MTHFR deficiency on sperm DNA methylation.

In keeping with a role for MTHFR in providing methyl groups for *de novo* DNA methylation in male germ cells, we found a predominance of DNA hypomethylation in the sperm of MTHFR-deficient males. The effect was consistent across the three MTHFR-deficient cohorts we examined: F1 fathers, F2 sons and the males from the Mat-Deficiency study. Remarkably, >80% of the sites affected in the sperm of the F2 sons overlapped with those affected in their fathers. In addition, similar biological pathways were affected. Together, the results suggest that there may be CpG sites that are consistently susceptible to germline MTHFR deficiency. Of note, and further supporting the link between MTHFR-deficiency and germ cell hypomethylation, there was little sperm hypermethylation associated with MTHFR deficiency along with little overlap in hypermethylated sites in sperm amongst the cohorts.

If epimutations accumulate across generations, we would have expected a higher number of hypomethylated sites and/or a more pronounced degree of hypomethylation in F2 versus F1 sperm. However, we observed fewer, lower magnitude, sperm DNA methylation changes in F2 generation MTHFR-deficient males compared with F1 generation MTHFR-deficient males. Thus, DNA methylation defects detected in sperm cannot explain the reproductive deterioration in the F2 generation. An alternative possibility is that more immature F2 male germ cells with the most abnormal DNA methylation pattern are lost early in germ cell development, an explanation consistent with the lower sperm count and increased abnormal testicular morphology in F2 sons compared with their F1 fathers.

We used published high-resolution sequencing data sets acquired from pure germ cell populations at different time points to uncover how and when CpG sites/regions affected in MTHFR-deficient sperm normally acquire DNA methylation during germ cell development. Interestingly, although most *de novo* DNA methylation patterns are established in male germ cells between E13.5 to E16.5, the regions that are sensitive to MTHFR loss in sperm colocalized with regions that usually acquire methylation at later stages of embryonic development (i.e. between E16.5 and P0). This observation is in keeping with our earlier study, in which we observed little effect of MTHFR deficiency on sites such as paternally methylated imprinted gene ICRs that are known to be methylated early (Chan et al., 2010). Consistent with their late-methylation status, at E16.5, MTHFR-sensitive sites were enriched for the histone modification H3K4me3, which inhibits DNMT3A/3L directed *de novo* DNA methylation (Ooi et al., 2007). High levels of H3K4me3 in the MTHFR-sensitive regions between E10.5 and E16.5 (Fig. 5C,D) also support the idea that these regions might be intrinsically refractory to early *de novo* DNA methylation in male germ cells. Further interrogation of H3K4me3 levels and DNA methylation in developing prenatal MTHFR-deficient germ cells is required to confirm this proposed sequence of events. Together, these results indicate that sequences subject to late methylation are selectively affected in MTHFR-deficient germ cells, with their enrichment for H3K4me3 suggesting a potential mechanism.

Our previous studies of the effects of F1 MTHFR deficiency on the development of embryonic and postnatal male germ cells support our findings here of a key defect in late embryonic DNA methylation events. F1 *Mthfr^−/−^* males on a BALB/c background have severely affected testes with a high percentage of abnormal seminiferous tubules and infertility as adults (Kelly et al., 2005). In these mice, although germ cell numbers are normal at E18.5 and P2, germ cell numbers start to decrease at P4 (by 15%) and are 70% lower by P6; there is a concomitant decrease in proliferation of germ cells at P4 and an increase in apoptosis at P6 (Kelly et al., 2003; Chan et al., 2010). Interestingly, administration of the dietary methyl donor betaine, which acts independently of the MTHFR pathway, has been shown to result in lower levels of apoptosis at P6, and improved testicular histology and fertility in *Mthfr^−/−^* males (Kelly et al., 2003). C57BL/6 strain *Mthfr^−/−^* mice show less-severe abnormalities in spermatogenesis than those seen in BALB/c mice and have normal germ cell numbers and germ cell proliferation perinatally (Chan et al., 2010). For both strains, detailed studies of adult spermatogenesis reveal heterogeneity among *Mthfr^−/−^* mice in testicular abnormalities; even within an individual testis, a variety of seminiferous tubule phenotypes are noted, from tubules with normal spermatogenesis to ones containing only Sertoli cells. Evidence of seminiferous tubule heterogeneity amongst adult *Mthfr^−/−^* males was also noted in the current study for both the F1 and F2 mice. Supported by our exogenous betaine administration experiments, it is possible that alternative sources of endogenous methyl donors may, in part, compensate for the MTHFR deficiency. Thus, the developmental studies indicate that the earliest evidence of phenotypic effects of MTHFR deficiency occur at the time prospermatogonia start to divide in the postnatal testis, between P2 and P3. The timing of appearance of the phenotype is similar to but less severe than that seen for mice with mutations in the germ-cell specific DNA methyltransferases 3C and 3L (Bourc'his et al., 2001; Barau et al., 2016).

What then are the characteristics of the late-methylated MTHFR-sensitive sites? More careful examination of the regions affected by MTHFR deficiency identified enrichment for young retrotransposons. TEs, which make up almost 40% of the mammalian genome, are important players in genome evolution (Goodier and Kazazian, 2008). Most TEs are not capable of retrotransposition, owing to the accumulation of mutations over time (Sookdeo et al., 2013). LINE-1 retrotransposons occupy almost 20% of the genome in human (Lander et al., 2001) and mouse (Waterston et al., 2002), and some younger families in both species still possess the ability to retrotranspose, causing germline and somatic mutations (Gagnier et al., 2019; Schauer et al., 2018). Germ cells use many strategies to suppress retrotransposon activity to maintain genome integrity, including DNA methylation, histone modifications and piwi-interacting RNA (piRNA)-mediated silencing (Kuramochi-Miyagawa et al., 2004). Intriguingly, MTHFR-sensitive regions were enriched in TEs (∼60%), with LTR (long terminal repeat) and LINE-1 family members showing the highest proportions. Most of the MTHFR-sensitive regions were found within or close to relatively young LINE-1 retrotransposons, specifically within the L1Md subfamily. Decreased DNA methylation in MTHFR-deficient germ cells could lead to abnormal expression of these young LINE-1 retrotransposons, which, in turn, could result in DNA damage, meiotic arrest and sterility (Soper et al., 2008; Yang and Wang, 2016; Zamudio and Bourc'His, 2010). Additional studies are needed to examine the expression levels of these young retrotransposons in MTHFR-deficient embryonic germ cells and test the correlation between loss of DNA methylation and expression from these MTHFR-sensitive regions.

The establishment of DNA methylation patterns during germ cell development is dependent on several factors. In a recent study, we demonstrated that NSD1, a histone lysine methyltransferase, deposits H3K36me2 marks in developing male germ cells to establish *de novo* DNA methylation patterns (Shirane et al., 2020). The *Nsd1* KO animals from this study show total lack of spermatogonia and display a more severe phenotype than DNMT3L-deficient males (Bourc'his et al., 2001; Shirane et al., 2020). Although DNA methylation at older LTRs and LINE-1 elements in the male germline is dependent on NSD1-mediated H3K36me2 deposition, a recently discovered member of the mouse DNMT family, DNMT3C, is required for DNA methylation silencing of active retrotransposons in the male germline, especially young LINE-1 elements and specific endogenous retrovirus group K members (ERVKs) in the male germline (Barau et al., 2016). It is therefore likely that one of these *de novo* DNA methylation pathways may be important for the acquisition of DNA methylation in the MTHFR-sensitive regions. By examining male germ cell DNA methylation patterns of these different KO mouse models, we revealed that the establishment of DNA methylation patterns at MTHFR-sensitive regions was largely dependent on DNMT3C, which is targeted specifically to young TEs, and on DNMT3L, which broadly influences deposition of this epigenetic mark. Using RNA-seq data from *Dnmt3c* KO P20 testis compared with WT (Barau et al., 2016), we identified that reactivated LINE-1 elements, which are normally methylated by DNMT3C, overlapped with >20% of all MTHFR-sensitive regions (±1 kb) and 46-60% of all young LINE-1 elements (Fig. 6B). Considering that DNMT3C only acts on a very specific subset of sites in the genome, it is interesting that MTHFR-sensitive regions seem to be particularly enriched in these DNMT3C-targeted sites. DNMT3C is also expressed at the highest levels in the late stages of embryonic germ cell development (peak expression at E16.5-E18.5), coinciding with the peak expression of MTHFR. In E16.5-E18.5 MTHFR-deficient germ cells, SAM levels, and thus availability of methyl groups for DNA methylation, may become limiting, providing a plausible explanation as to why DNMT3C- and DNMT3L-dependent late-methylated regions might be sensitive to MTHFR deficiency.

In addition to providing a potential explanation for the abnormal testis histology, hypomethylation of retrotransposons could also explain deterioration in reproductive parameters across generations (Fig. 6C). We propose that hypomethylation of young retroelements in the sperm of MTHFR-deficient F1 males may persist during the DNA re-methylation phase in peri-implantation stage F2 males, and that such hypomethylated retrotransposons will also be present in the developing germ cells of the F2. Abnormal hypomethylation of young retrotransposons in the early germline of the F2 MTHFR-deficient males provides a plausible mechanism for the increasingly abnormal testicular phenotypes across generations. Together, the worsening of reproductive parameters in MTHFR-deficient sons versus their fathers and the profound hypomethylation of sperm DNA, particularly at evolutionarily young retrotransposons, suggest that epigenetic defects may accumulate across generations, findings consistent with the intergenerational inheritance of epimutations. An important limitation of our study is that we did not confirm a causal link between hypomethylation of young retrotransposons and the reproductive phenotype observed. Owing to the potential complexity of the mechanism involved, follow-up studies will require two-generation breeding (F1, F2) of *Mthfr^−/−^* males with GFP-marked germ cells, fluorescence-activated cell sorting of embryonic germ cells before (PGCs) and after (prospermatogonia) expression of MTHFR as well as post-replication (spermatogonia) in the postnatal testis, along with DNA methylome, transcriptome and histone methylome studies on multiple germ cell types to determine the sequence of events.

## MATERIALS AND METHODS

### Animals

All animal work was handled in accordance with the Canadian Council on Animal Care guidelines. Approvals were received from the Facility Animal Care Committee (FACC) at the Research Institute of the McGill University Health Centre in Montreal for the F1 and F2 generations and the Animal Care Committee of the University of Ottawa Faculty of Medicine for the maternal-deficient cohort. *Mthfr^+/+^*, *Mthfr^+/−^* and *Mthfr^−/−^* C57BL/6 mice (backcrossed to Charles River C57BL/6 background for at least ten generations) were kept in temperature-controlled (18-24°C), pathogen-free rooms with 12 h light 12 h dark cycle and had free access to water and Teklad Global 18% or 19% Protein Rodent Diet (Envigo), which contains 4 mg/kg folate and 1200 mg/kg choline supplementation. *Mthfr^+/−^* female mice used to produce the F2 generation of *Mthfr^−/−^* males were derived from heterozygous breedings. Both *Mthfr^+/−^* and *Mthfr^−/−^* females produced litters. The *Mthfr* genotypes were determined by a previously described PCR-based method (Chen et al., 2001). Mice were sacrificed, their reproductive organs removed and weighed, and mature sperm from paired cauda-epididymides were collected. All tissue and sperm samples were stored at −80°C until further use.

### Hemacytometric sperm counts

A weighed part of the frozen testis was homogenized (Polytron, Brinkmann Instruments) for 2×15 s inside a 5 ml homogenizing solution (0.9% NaCl, 0.1% thimerosal and 0.5% Tween-20) and spermatozoal heads, which are resistant to homogenization, were counted as described by Kelly et al. (2003)*.*

### Testicular histology analysis

Freshly collected and cleaned testes were fixed with Bouin's Fixative solution (BDH) overnight, followed by immersion in 70% ethyl alcohol. Fixed testes were cut in half and embedded in paraffin, cut into 5-µm-thick serial sections and every sixth section was stained with Hematoxylin and Eosin (H&E). Four to eight sections (each section with at least 100 tubules) from each animal were examined under a Zeiss Axiophot compound microscope and imaged using a AxioCamMRc camera with Axiovision v4.7.1.0 software (Carl Zeiss). Normal tubules were identified as having all germ cell types in the majority of the seminiferous epithelium but could contain some mild alterations (e.g. small and few vacuoles). Abnormal tubules were identified as possessing at least one of the following criteria: (1) asymmetric distribution of germs cells within the tubule, germ cells present in a part of the tubule cross-section but not on the other part; (2) tubule with early germ cells but missing spermatids; (3) tubule with spermatozoa and spermatids but no apparent early germ cells; (4) Sertoli-cell only phenotype: tubule has only Sertoli cells, no noticeable germ cells within the tubule. The number of abnormal tubules in 100 seminiferous tubules examined for each mouse was used to calculate the proportion of abnormal tubules.

### DNA isolation from sperm

Sperm was pretreated overnight at 37°C with a lysis buffer containing EDTA, Tris, sarkosyl, dithiothreitol and proteinase K. The lysate was used for DNA isolation with DNeasy Blood & Tissue Kit (QIAGEN) according to the manufacturer's protocol.

### Reduced representation bisulfite sequencing (RRBS)

RRBS libraries were prepared following previously published protocols using the gel-free technique (Boyle et al., 2012; Gu et al., 2011; Mcgraw et al., 2015) with minor modifications (Rahimi et al., 2019). Briefly, 700 ng of sperm DNA from each sample was digested with MspI (New England Biolabs, NEB) overnight. MspI-digested DNA samples were end-repaired and A-tailed using Klenow fragment (NEB). Agencourt AMPure XP magnetic beads (Beckman Coulter) were used to clean up the DNA and cleaned DNA was used for Methylated Adaptors (NEB) ligation. Following ligation, two rounds of bisulfite conversion were performed on the DNA samples using the EpiTect Bisulfite kit (QIAGEN) following the manufacturer's instructions. qPCR was performed with the addition of 1 µl of SYBR Green Nucleic Acid Stain 5X (Invitrogen) to the regular PCR reaction using the Lightcycler 96 System (Roche) in order to determine the optimal cycle for large-scale qPCR. Large-scale qPCR was performed with previously determined cycle numbers and followed by additional rounds of bead clean-up.

Prepared RRBS libraries were sent to the McGill University and Genome Quebec Innovation Centre (Montreal, QC, Canada). Twelve samples were multiplexed and were sequenced in one lane with paired end sequencing on a Hi-Seq 2000 sequencer (Illumina). Raw data were processed and aligned using bsmap (version 2.74) (Xi and Li, 2009). Differential methylation was determined using MethylKit software (version 1.1) (Akalin et al., 2012). MethylKit software uses the Benjamini–Hochberg false discovery (FDR)-based method for *P*-value correction (q=0.01). One hundred bp stepwise tiling-windows were used for the analysis, each window containing a minimum of two CpGs with 10× coverage (or sequencing depth) and 10% or more difference between groups. Tiles fitting the above-mentioned criteria were considered as DMTs and were annotated with HOMER software (version 4.9.1) (Heinz et al., 2010). Through MethylKit, DMCs were identified as CpGs having 10× coverage in both groups with a difference of at least 10%. Significant DMCs within close proximity (100 bp) were merged using the merge function of bedtools (version 2.29.0) to determine DMRs. Similarly, the intersection of DMTs and overlapping DMRs was determined using the intersectBed function. GO enrichment analysis using genic DMTs was performed against the all sequenced genic tiles list (as background) using a web-based functional enrichment analysis tool, WebGestalt (WEB-based GEne SeT AnaLysis Toolkit) (Liao et al., 2019).

### Statistical analyses

GraphPad Prism 6.0e (GraphPad Software) was used for generating graphs and statistical analysis. Significance threshold was set to *P*<0.05 for all the tests. Continuous variables were tested with Student's *t*-test (unpaired, two-tailed) and categorical variables were tested with χ^2^ test.

### Analysis of publicly available datasets

Publicly available datasets were re-processed as described previously (Shirane et al., 2020). WGBS data were derived from E13.5 male PGCs, E16.5 PSG (Kobayashi et al., 2013), P0 PSG, spermatozoa (Kubo et al., 2015), spermatocyte (Hammoud et al., 2014), *Nsd1* HET and KO P0 PSG (Shirane et al., 2020) and *Dnmt3c* and *Dnmt3l* KO P10 germ cells with matching controls (Barau et al., 2016). H3K4me3 ChIP-seq data were derived from E10.5 and E13.5 male PGCs (Kawabata et al., 2019), E16.5 and P0 PSG (Shirane et al., 2020), spermatocyte (Baker et al., 2014) and spermatozoa (Erkek et al., 2013). RNA-seq data were derived from *Dnmt3c* and *Dnmt3l* KO P10 germ cells with matching controls (Barau et al., 2016).

## Supplementary Material

Supplementary information

Reviewer comments
