# Supplementary material for: Paternal MTHFR deficiency leads to hypomethylation of young retrotransposons and reproductive decline across two successive generations
Source: Development. 2021 Jul 6;148(13):dev199492. doi: 10.1242/dev.199492 (PMC8276981; doi:10.1242/dev.199492)
Supplement: Supplementary information [file develop-148-199492-s1.pdf]

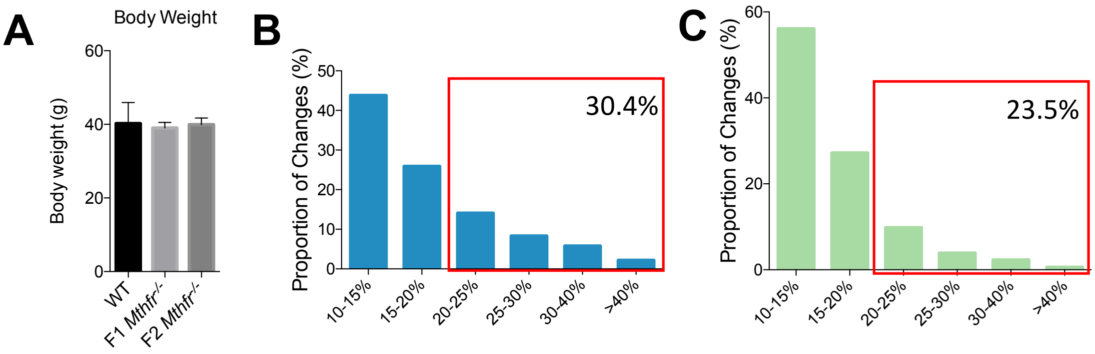

Figure S1. Genome wide sperm DNA methylation analysis of DMTs in MTHFR deficient F1 and F2 generations. (A) Body weights of WT and MTHFR deficient C57BL/6 mice. Proportion of magnitude of changes in the sperm DNA methylation of DMTs in (B) F1 generation, (C) F2 generation MTHFR deficient groups compared to WT.

Development • Supplementary information

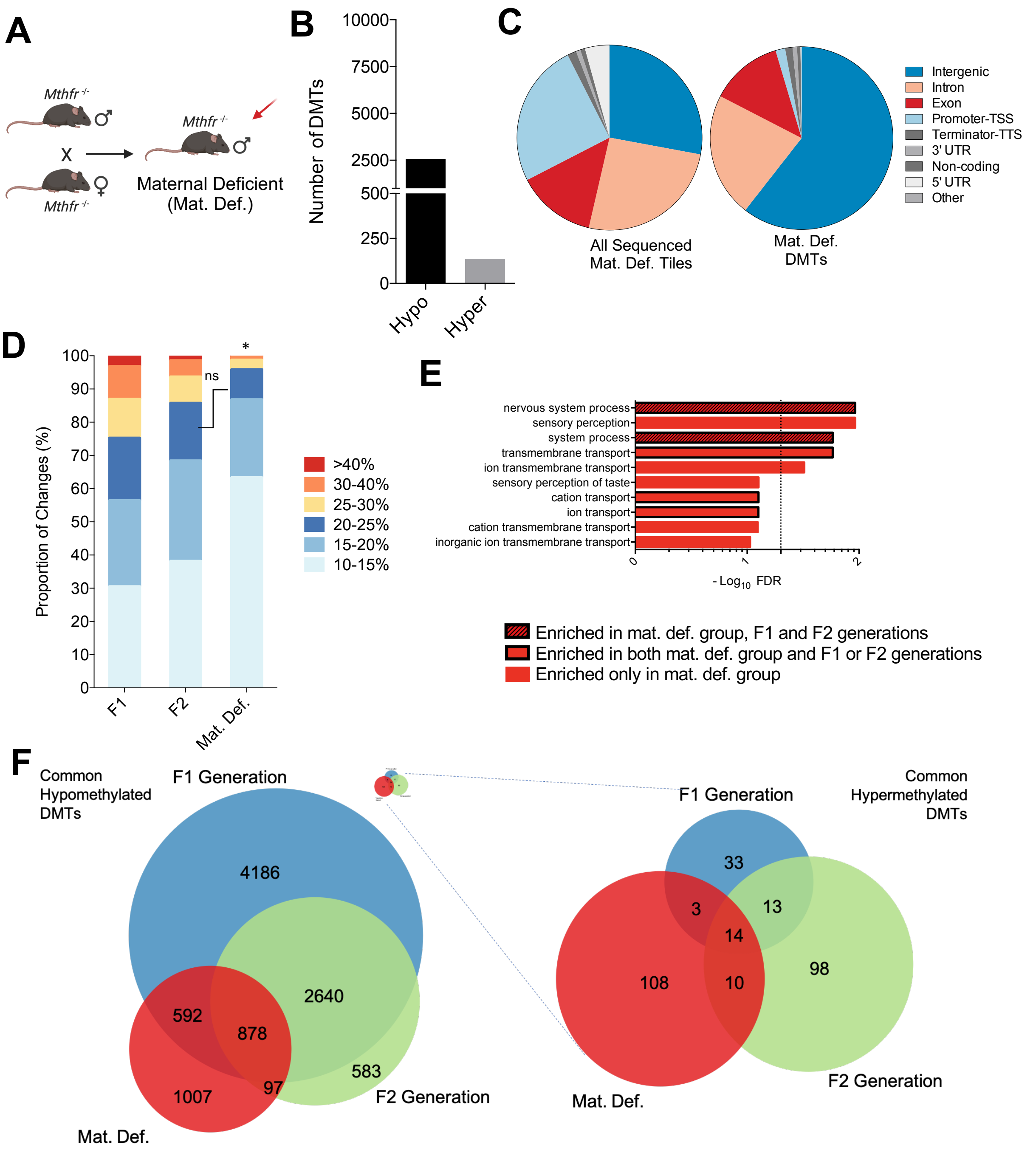

Figure S2. Genome-wide sperm DNA methylation analysis of maternal deficient (Mat. Def.) *Mthfr* $^{-/-}$ males. (A) Breeding scheme for Mat. Def. group. (B) Number of 100bp tiles that significantly hypomethylated or hypermethylated in Mat. Def. group compared to their respective WT group. (C) Distribution of Mat. Def. group DMTs and all sequenced Mat. Def. tiles into genomic elements are shown in pie chart. (D) Proportion of magnitude of changes in F1, F2 generations in comparison to Mat. Def. group. If not shown as not significant (ns), p<0.05; *P*-values were calculated by Chi-square test. (E) Gene Ontology enrichment analysis of genic differentially methylated tiles (DMTs) in Mat. Def. group, darker bars and boxed bars indicate the degree of the common enriched pathways between Mat. Def. group and F1, F2 generations. The dotted line indicates p<0.05 threshold for significance for false discovery rate (FDR). (F) Euler diagrams of common hypo- and hypermethylated tiles between Mat. Def. group, F1 and F2 generations. Hypermethylated tiles were shown proportional (in size) to the hypomethylated tiles on top right of the common hypomethylated DMTs graph and magnified version was given on the right.

Development • Supplementary information

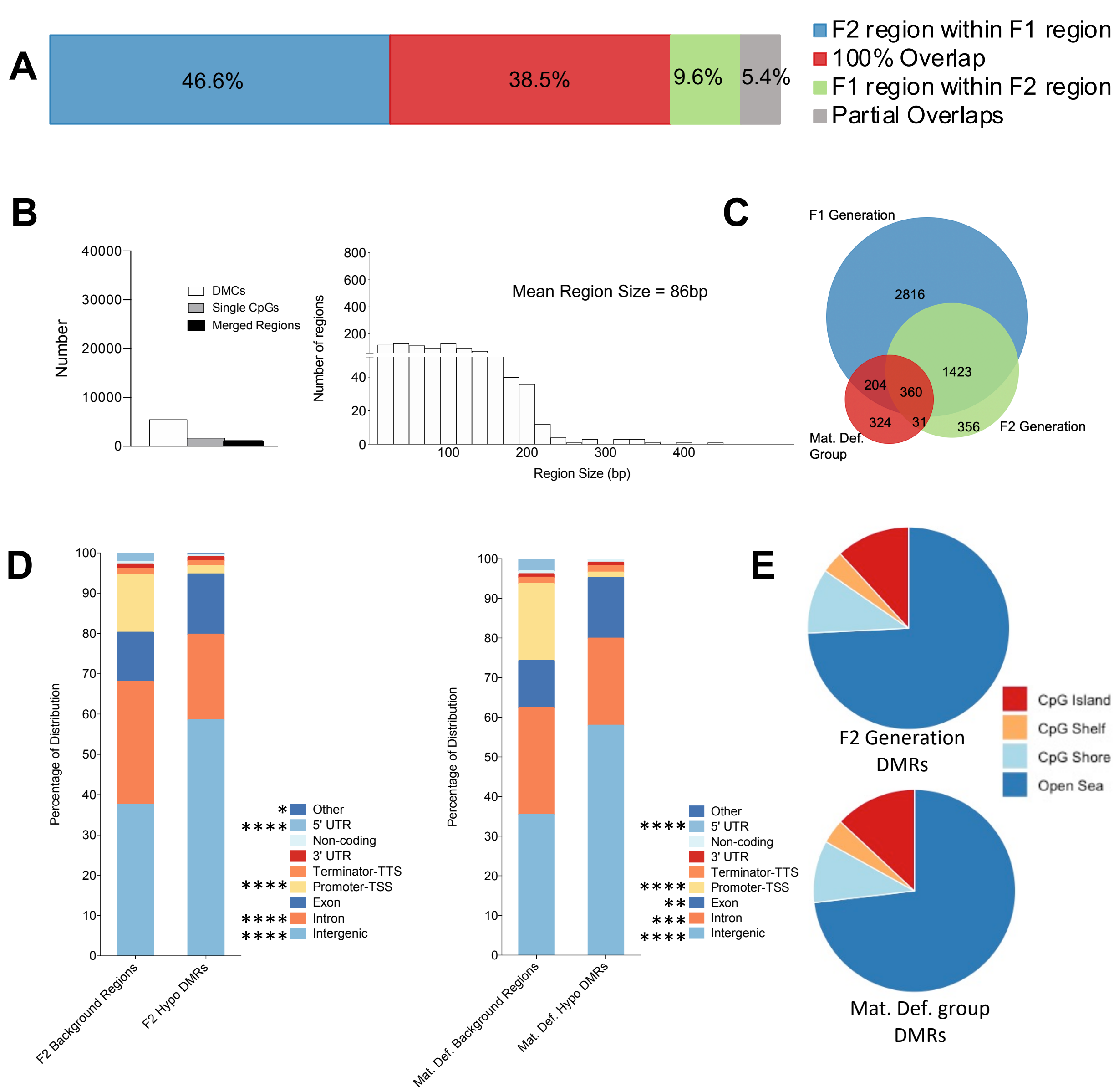

Figure S3. DMR analysis and comparison in all three groups of MTHFR-deficient male mice. (A) Distribution of common F1 and F2 hypomethylated merged regions according to their degree of overlap with each other. (B) Total number of hypomethylated merged regions in Mat. Def. group are compared with all DMCs and single CpGs on the left and the distribution of these merged regions according to their sizes are shown on the right, graphs are scaled. (C) Euler diagram of common hypomethylated merged regions between F1, F2 generation and Mat. Def. group. (D) Distribution of F2 and Mat. Def. merged hypomethylated DMRs into genomic elements in comparison to all sequenced merged F2 and Mat. Def. regions, respectively; ****, $p<0.0001$; ***, $p<0.001$; **, $p<0.01$ and *, $p<0.05$; *P*-values were calculated by Chi-square test. (E) Location of F2 and Mat. Def. group merged hypomethylated DMRs with regards to CpG islands/shores/shelves and open sea regions

Development • Supplementary information

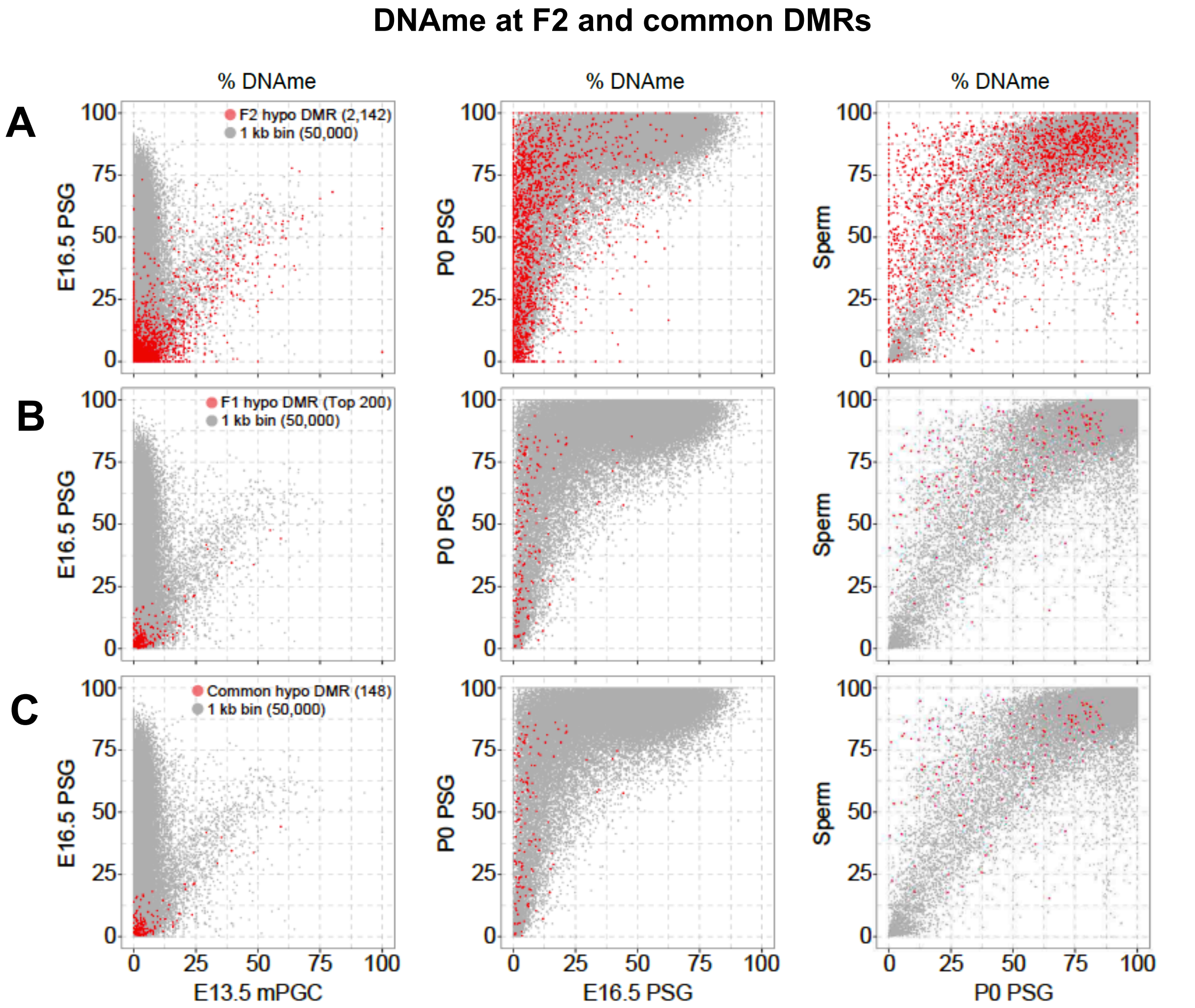

Figure S4. DNA methylation (DNAme) dynamics at F2 and common DMRs during spermatogenesis compared to whole genome. Scatterplots showing % DNAme in (A) F2 hypomethylated DMRs, (B) F1 hypomethylated Top 200 DMRs (by size) and (C) Common DMRs between F2 hypomethylated DMRs and F1 hypomethylated Top 200 DMRs (red dots), compared to whole genome 1kb bins (gray dots) at E13.5 (Kobayashi et al. 2013), E16.5, P0 (Shirane et al., in press) and sperm (Kubo et al. 2015).

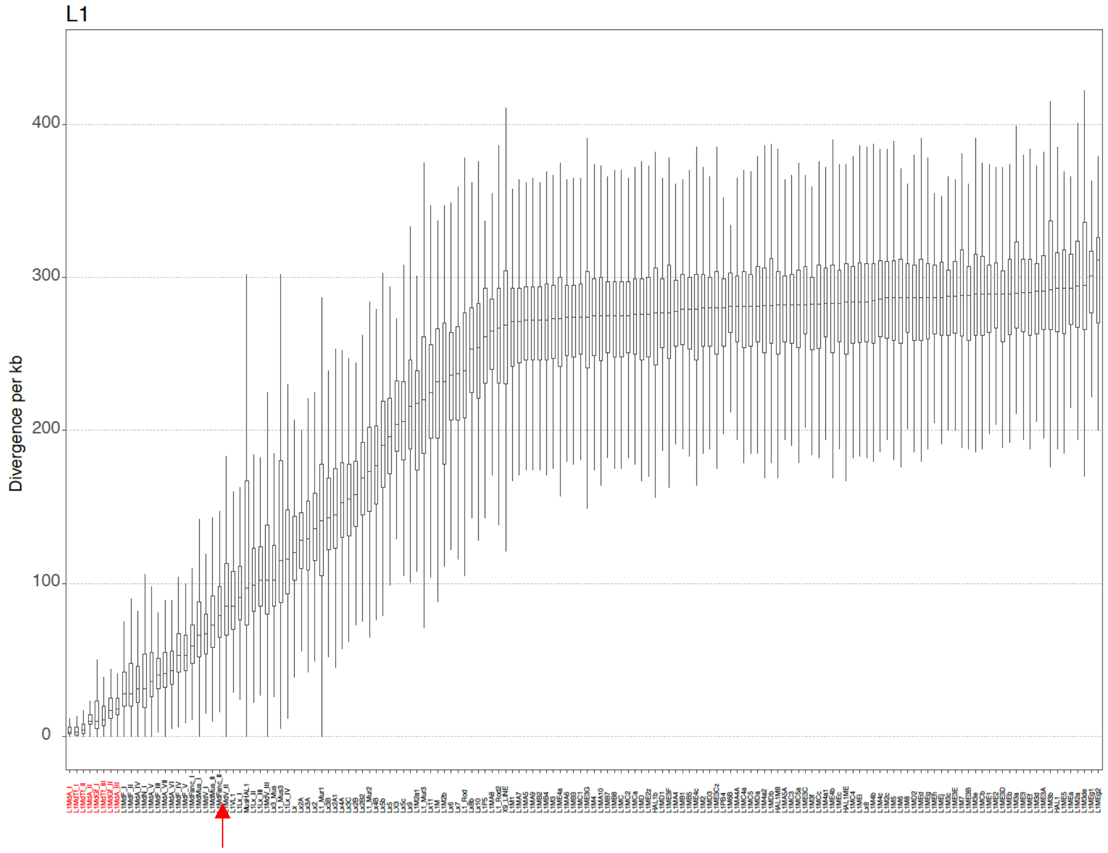

Figure S5. Identification of young LINEs according to their divergence. Red arrow indicates the starting point of LINE elements considered young (left side of the arrow). LINE subfamilies written in red show the DNMT3C sensitive young LINEs, that are identifies as having a >5-fold difference in expression in *Dnmt3c*$^{-/-}$ compared to *Dnmt3c*$^{+/-}$ in P20 testis (Barau et al. 2016).

Development • Supplementary information

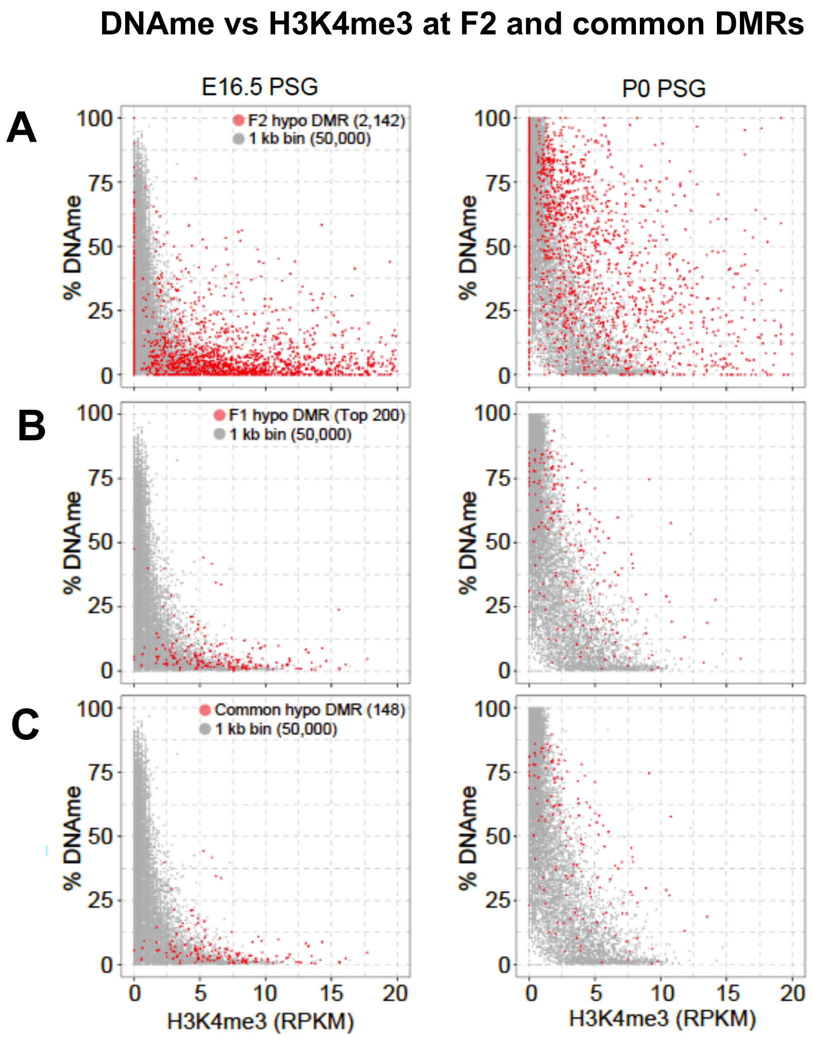

Figure S6. The changes of DNA methylation and H3K4me3 occupancy on MTHFR sensitive F2 hypomethylated and common DMRs during spermatogenesis compared to whole genome. Scatterplots of %DNAme and H3K4me3 levels at (A) F2 hypomethylated DMRs, (B) F1 hypomethylated Top 200 DMRs (by size) and (C) Common DMRs between F2 hypomethylated DMRs and F1 hypomethylated Top 200 DMRs (red dots), compared to whole genome 1kb bins (gray dots) at E16.5 and P0 (Shirane et al., in press). RPKM, Reads Per Kilobase Million.

Development • Supplementary information

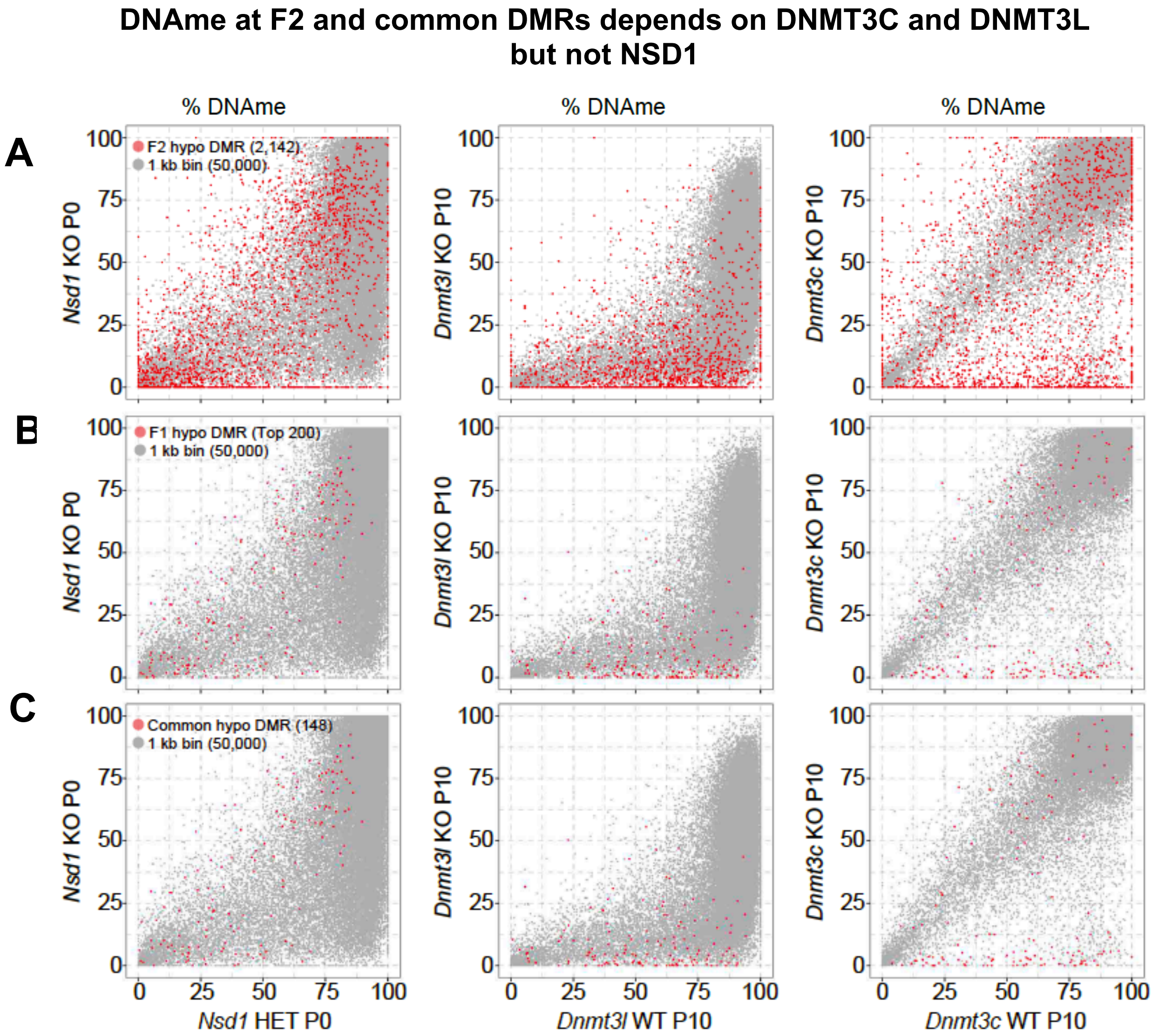

Figure S7. The effect of epigenetic regulators on DNA methylation dynamics of F2 and common MTHFR sensitive DMRs. The effect of NSD1 (Shirane et al., in press) DNMT3L and DNMT3C deficiency (Barau et al. 2016) on DNA methylation levels at MTHFR sensitive (A) F2 hypomethylated DMRs, (B) F1 hypomethylated Top 200 DMRs (by size) and (C) Common DMRs between F2 hypomethylated DMRs and F1 hypomethylated Top 200 DMRs (red dots), compared to whole genome 1kb bins (gray dots).

Development • Supplementary information

Table S1. Number of DMTs identified in MTHFR deficient sperm compared to WT groups

	Hypomethylated	Hypermethylated	Total
F1 Generation *Mthfr* $^{-/-}$	8296	63	8359
F2 Generation *Mthfr* $^{-/-}$	4198	134	4332
Mat. Def. Group *Mthfr* $^{-/-}$	2574	135	2709

Supplementary References

Baker, Christopher L., Michael Walker, Shimpei Kajita, Petko M. Petkov, and Kenneth Paigen. 2014. "PRDM9 Binding Organizes Hotspot Nucleosomes and Limits Holliday Junction Migration." *Genome Research* 24 (5): 724–32. https://doi.org/10.1101/gr.170167.113.

Barau, Joan, Aurélie Teissandier, Natasha Zamudio, Stéphanie Roy, Valérie Nalesso, Yann Hérault, Florian Guillou, and Déborah Bourc'his. 2016. "The DNA Methyltransferase DNMT3C Protects Male Germ Cells from Transposon Activity." *Science* 354 (6314): 909–12. https://doi.org/10.1126/science.aah5143.

Erkek, Serap, Mizue Hisano, Ching Yeu Liang, Mark Gill, Rabih Murr, Jürgen Dieker, Dirk Schübeler, Johan Van Der Vlag, Michael B. Stadler, and Antoine H.F.M. Peters. 2013. "Molecular Determinants of Nucleosome Retention at CpG-Rich Sequences in Mouse Spermatozoa." *Nature Structural and Molecular Biology* 20 (7): 868–75. https://doi.org/10.1038/nsmb.2599.

Hammoud, Saher Sue, Diana H.P. Low, Chongil Yi, Douglas T. Carrell, Ernesto Guccione, and Bradley R. Cairns. 2014. "Chromatin and Transcription Transitions of Mammalian Adult Germline Stem Cells and Spermatogenesis." *Cell Stem Cell* 15 (2): 239–53. https://doi.org/10.1016/j.stem.2014.04.006.

Kawabata, Yukiko, Asuka Kamio, Yuko Jincho, Akihiko Sakashita, Tomoya Takashima, Hisato Kobayashi, Yasuhisa Matsui, and Tomohiro Kono. 2019. "Sex-Specific Histone Modifications in Mouse Fetal and Neonatal Germ Cells." *Epigenomics* 11 (5): 543–61. https://doi.org/10.2217/epi-2018-0193.

Kobayashi, Hisato, Takayuki Sakurai, Fumihito Miura, Misaki Imai, Kentaro Mochiduki, Eikichi Yanagisawa, Akihiko Sakashita, et al. 2013. "High-Resolution DNA Methylome Analysis of Primordial Germ Cells Identifies Gender-Specific Reprogramming in Mice." *Genome Research* 23 (4): 616–27. https://doi.org/10.1101/gr.148023.112.

Kubo, Naoki, Hidehiro Toh, Kenjiro Shirane, Takayuki Shirakawa, Hisato Kobayashi, Tetsuya Sato, Hidetoshi Sone, et al. 2015. "DNA Methylation and Gene Expression Dynamics during Spermatogonial Stem Cell Differentiation in the Early Postnatal Mouse Testis." *BMC Genomics* 16 (1): 624. https://doi.org/10.1186/s12864-015-1833-5.
