# Supplementary material for: Paternal MTHFR deficiency leads to hypomethylation of young retrotransposons and reproductive decline across two successive generations
Source: Development. 2021 Jul 6;148(13):dev199492. doi: 10.1242/dev.199492 (PMC8276981; doi:10.1242/dev.199492)
Supplement: Reviewer comments [file develop-148-199492_review_history.pdf]

Paternal MTHFR deficiency leads to hypomethylation of young retrotransposons and reproductive decline across two successive generations

Gurbet Karahan, Donovan Chan, Kenjiro Shirane, Taylor McClatchie, Sanne Janssen, Jay M. Baltz, Matthew Lorincz and Jacquetta Trasler
DOI: 10.1242/dev.199492

Editor: Haruhiko Koseki

Review timeline
Original submission: 3 February 2021
Editorial decision: 11 March 2021
First revision received: 10 May 2021
Accepted: 1 June 2021

Original submission

First decision letter

MS ID#: DEVELOP/2021/199492

MS TITLE: Paternal MTHFR deficiency leads to reproductive decline across generations in association with hypomethylation of young retrotransposons

AUTHORS: Gurbet Karahan, Donovan Chan, Kenjiro Shirane, Taylor McClatchie, Sanne Janssen, Jay M. Baltz, Matthew Lorincz, and Jacquetta Trasler

I have now received all the referees' reports on the above manuscript, and have reached a decision. The referees' comments are appended below, or you can access them online: please go to BenchPress and click on the 'Manuscripts with Decisions' queue in the Author Area.

As you will see, the referees express considerable interest in your work, but have some significant criticisms and recommend a substantial revision of your manuscript before we can consider publication. If you are able to revise the manuscript along the lines suggested, which may involve further experiments, I will be happy receive a revised version of the manuscript. Your revised paper will be re-reviewed by one or more of the original referees, and acceptance of your manuscript will depend on your addressing satisfactorily the reviewers' major concerns. Please also note that Development will normally permit only one round of major revision.

We are aware that you may be experiencing disruption to the normal running of your lab that make experimental revisions challenging. If it would be helpful, we encourage you to contact us to discuss your revision in greater detail. Please send us a point-by-point response indicating where you are able to address concerns raised (either experimentally or by changes to the text) and where you will not be able to do so within the normal timeframe of a revision. We will then provide further guidance. Please also note that we are happy to extend revision timeframes as necessary.

Please attend to all of the reviewers' comments and ensure that you clearly highlight all changes made in the revised manuscript. Please avoid using 'Tracked changes' in Word files as these are lost in PDF conversion. I should be grateful if you would also provide a point-by-point response detailing how you have dealt with the points raised by the reviewers in the 'Response to Reviewers' box. If you do not agree with any of their criticisms or suggestions please explain clearly why this is so.

© 2021. Published by The Company of Biologists under the terms of the Creative Commons Attribution License (https://creativecommons.org/licenses/by/4.0/).

Reviewer 1

Advance summary and potential significance to field

In this manuscript, Karahan and colleagues investigate the consequences of two generations of paternal MTHRF deficiency on the male germline. They show that absence of MTHRF, which is crucial in generating methyl donor groups, results in reduced fertility in the F1 generation and infertility in F2 males. The investigators also analyze DNA methylation in mature sperm, revealing a large number of hypomethylated sequences, although more differential methylation is found in the F1 sperm. Analysis of sequences show preferential demethylation of retrotransposons. Additionally, mining of previously published data indicates that sequences subject to late methylation are selectively affected in MTHFR-deficient germ cells. Moreover these sequences are enriched for H3K4me3 suggesting a potential mechanism for targeted methylation in the male germline. Together, the results are interesting and important, revealing new insights into biology of reprogramming of the male germline.

Comments for the author

This work is very nicely carried out and described. It is also impressive to see how the investigators mined data to back up their observations that DNA demethylated regions are enriched for young retrotransposons (LINE-1 elements), which are targets of DNMT3C. One experiment that would have been important to see potentially underscoring the hypothesis that activation of their repetitive elements in the germline may be associated with diminished germ cells, would be to assay young LINE-1 transcription late in gestation.

Moreover, given the surprising observation that there are more methylation defects in sperm from F1 vs F2 mice, and the hypothesis that more adversely affected germ cells are lost in F2 mice, it seems that a comparison of transcription in prospermatogonia in F1 and F2 null mice.

Specific (largely minor) comments:

Abstract: “late prenatal germ cell development”. The term prenatal is used a lot in the manuscript but in the abstract, it seems more difficult for this reader to process. How about late fetal or embryonic? This is a bit more intuitive for a developmental biologist.

Introduction, lines 68-80. In this paragraph, the authors discuss the DNMT enzymatic domains, including the PWWP and ADD domains. Although it is stated what histones these domains interact with, no information is given as to why the interaction is significant. Perhaps an extra sentence would be informative.

Figure 1b: the testis weight and sperm count are clearly lower in F1 null males but not statistically significant.

This is because of the small number of animals assayed (3,4). Is it possible to increase number slightly?

With respect to the production of F2 null males, Mthfr +/- females were used. How are these animals derived (heterozygous matings)? Is it known if these females have any defects in DNA methylation in their germline?

Reviewer 2

Advance summary and potential significance to field

In this interesting manuscript the authors provide evidence that paternal deficiency (with or without maternal deficiency) of methylenetetrahydrofolate reductase (MTHFR) can lead to male reproductive abnormalities across two generations. The focus of the work reported is not so much on the reproductive abnormalities as it is on genomic analysis of epimutations (hypomethylation), postulated to be a transmission mechanism of the reproductive defects. The authors investigated

© 2021. Published by The Company of Biologists under the terms of the Creative Commons Attribution License (https://creativecommons.org/licenses/by/4.0/).

wild-type parameters of the DNA-methylation (DNAme) sites sensitive to absence of MTHFR. Both generations of deficient males show considerable loss of sperm DNAme in overlapping regions that are enriched in young retrotransposons. Importantly, these sites appear to be methylated at the time of expression of MTHFR in prenatal male germ cells. Although the findings are intriguing, the authors did not conduct experiments to tie genomic DNAme depletion to the reproductive phenotype and thus the results lack mechanistic significance.

Comments for the author

This manuscript is well written and a pleasure to read. The organization is a logical progression through the work. The figures are well organized and laid out, easily interpretable without resorting to the legends, which are nonetheless clear. The findings will be of interest to the community of developmental biologists.

For the most part, the conclusions are supported by the data. One area where this deserves more careful attention is in the last section of Results, with respect to Fig. 6A. Evaluation of these three figure panels by “eyeball statistics” suggests that all three proteins, NSD1, DNMT3L and DNMT3C, play a role in methylation of the MTHFR-sensitive sites, with greatest effect exerted by DNMT3L. Elaboration on the conclusion that DNA methylation at these sites depends on DNMT3L and DNMT3C but not NSD1 would be valuable, as would justification for follow-up on Dnmt3c KO data rather than data from Dnmt3l KOs, where the effect was seemingly the greatest.

There is one overriding concern about the focus of this manuscript. In spite of the title of the manuscript, most of the emphasis is on the genomic distribution of DNAme marks (potential epimutations) and how they change in absence of MTHFR. Relatively little attention is given to the phenotype (less than 1 figure of a total of 6 figures). Thus, interesting though they may be, the genomic findings have not been tied mechanistically to the reproductive phenotype except by speculation. Moreover, if the epimutations are responsible for the phenotype, it is puzzling that they are fewer DMTs in F2 sperm than in F1 sperm. As the authors point out, this could be explained by loss of affected germ cells early in their development, but this could be bolstered more extensive analysis of germ-cell death. In summary, while the body of the genomic analysis is impressive, the impact of the genomic findings would be greatly enhanced if they could be better tied to the reproductive defects. For example, determination if LINE 1 elements are or are not mis-expressed in mutant germ cells would inform if the simplest inferred relationship is valid or if the mechanisms are more complex.

<u>Reviewer 3</u>

Advance summary and potential significance to field

In the manuscript “Paternal MTHFR deficiency leads to reproductive decline across generations in association with hypomethylation of young retrotransposons”, Karahan et al show that the loss of 5, 10-Methylenetetrahydrofolate reductase (MTHFR) leads to widespread hypomethylation of the sperm genome in F1 and F2 generations. Intriguingly, however, reproductive defect is exacerbated in the F2 mice, while the F1 mice are fertile. Based on these results, the authors propose that decrease of DNA methylation in MTHFR-/- mice causes reproductive decline across generations.

Comments for the author

The Comments for the authors are given below.

1. The authors show reproductive defects specifically in the F2 generation, which is slightly different from reproductive decline taking place across successive generations. As such, it might be better to modify the title so that it represents the results better.

2. The F2 phenotype shown here could also indicate primordial germ cells defects during gestation. Indeed, the authors (Niles et al, PLoS one, 2011) previously reported a similar finding. In the present manuscript, the authors also note that “Based on high levels of expression of MTHFR in prospermatogonia (PSG), MTHFR deficiency is expected to affect F1 generation (1st hit) primordial

© 2021. Published by The Company of Biologists under the terms of the Creative Commons Attribution License (https://creativecommons.org/licenses/by/4.0/).

germ cells (PGCs) when DNA methylation patterns are established" (lines 683-686). Potential aberrations in the PGC methylome should be analyzed for better interpretation of the results.

3. The reproductive defect discussed in this paper is mostly based on sperm count and testis weight. A more detailed analysis should be required (for example, see Dong et al, Nat Commun, 2019; PMID: 31624244). At which stage(s) of spermatogenesis/spermiogenesis do these defects occur?

4. The authors propose that aberrant de-repression of retrotransposable elements caused by hypomethylation of the genome, leads to reproductive defects in F2 mice. This claim should be backed up with RNA-seq data showing the actual de-repression of the repeats. Measuring the level of piRNAs (see Dong et al, Nat Commun, 2019) may also be performed.

Taken together, the results shown in this paper are certainly interesting. However, the mechanistic explanation of why F2 mice show enhanced reproductive defects needs to be more rigorously explored.

First revision

Author response to reviewers' comments

Response to Editor and Reviewers: General Comments

We thank the Reviewers for their suggestions and provide a point-by-point response to the individual comments below. In this preamble, we would like to address head-on what would be required to respond to the suggestions of the Reviewers for additional experiments that would help confirm our proposed mechanism for the more severe phenotype in the F2 versus the F1 males. We agree that such experiments are important next steps. However, we think that the current study stands on its own in its detailed characterization of DNA hypomethylation in MTHFR-deficient mice, the identification of young retrotransposons as commonly affected sites across 2 generations and an exploration of the developmental mechanisms (via mining of embryonic and early postnatal male germ cell datasets) underlying the male germ cell DNA methylation defects. To confirm a causal link between hypomethylation of young retrotransposons and the reproductive decline in F2 versus F1 MTHFR-deficient males, would take at least one year (without considering COVID-19 slowdowns), with studies requiring two generation breeding (F1, F2), early germ cell isolations, and multi-omics on small numbers of cells.

To summarize, what we hypothesize is that: 1) as a result of MTHFR deficiency (occurring in germ cells starting at ~E15-18), while F1 PGCs show normal DNA methylation, F1 spermatogonia and sperm are DNA demethylated at key sequences including young retrotransposons, 2) the demethylated young retrotransposons escape remethylation in early peri-implantation embryos and thus F2 PGCs carry demethylation of young retrotransposons into the early germline (the step when inheritance of epimutations occurs- the most intriguing aspect of the MTHFR-deficiency model), 3) together, the demethylation of young retrotransposons in F2 PGCs (could perturb gene expression or other events) and the F2 germline MTHFR deficiency (again starting at ~E15-18) contribute to the lower sperm counts and/or deterioration in testicular histology in the F2 adult males.

To test our hypothesis would require us to: 1) retrieve frozen sperm from an Oct4-GFP line back crossed into the same C57B6 background as the *Mthfr*$^{+/-}$ mice (necessary to have same background as the subfertility phenotype is lost on a mixed background), 2) produce sufficient adult *Mthfr*$^{+/-}$ males, 3) cross Oct4-GFP and *Mthfr*$^{+/-}$ mice, 4) from *Mthfr*$^{+/-}$Oct4GFP parents, produce *Mthfr*$^{-/-}$Oct4-GFP males from which to collect PGCs at E13.5 (before MTHFR is expressed in male germ cells), prospermatogonia at postnatal day 0 (after MTHFR is expressed but before replication in spermatogonia- time of cell loss in earlier studies), and sperm. DNA

© 2021. Published by The Company of Biologists under the terms of the Creative Commons Attribution License (https://creativecommons.org/licenses/by/4.0/).

methylome, ChIP-Seq and RNA-Seq would be needed on the isolated germ cells, 5). Similar experiments would need to be done in the F2 generation- i.e examination of PGCs, E15-E18 prospermatogonia, spermatogonia and sperm.

After careful consideration, we think the current study is complete and suggest that it is unlikely we could provide meaningful mechanistic information regarding the proposed inheritance of epimutations in the F2 without thorough examination of multiple isolated male germ cell types (PGCs, prospermatogonia, spermatogonia, sperm) from both the F1 and F2 generation. As young retrotransposons are demethylated in F1 and F2 sperm, increased expression of such sequences in one germ cell type may not provide mechanistic insight. As MTHFR is involved in both histone and DNA methylation, both processes will need to be examined in detail in a developmental context.

We have added text to the Discussion indicating the limitation of our study re-deciphering the underlying mechanism and outlining the experiments that would need to be done (lines 566-574).

Response to Individual Reviewers (changes to the original manuscript are marked in yellow)

Reviewer 1 Advance Summary and Potential Significance to Field:
In this manuscript, Karahan and colleagues investigate the consequences of two generations of paternal MTHRF deficiency on the male germline. They show that absence of MTHRF, which is crucial in generating methyl donor groups, results in reduced fertility in the F1 generation and infertility in F2 males. The investigators also analyze DNA methylation in mature sperm, revealing a large number of hypomethylated sequences, although more differential methylation is found in the F1 sperm. Analysis of sequences show preferential demethylation of retrotransposons. Additionally, mining of previously published data indicates that sequences subject to late methylation are selectively affected in MTHFR-deficient germ cells. Moreover, these sequences are enriched for H3K4me3 suggesting a potential mechanism for targeted methylation in the male germline. Together, the results are interesting and important, revealing new insights into biology of reprogramming of the male germline.

Reviewer 1 Comments for the Author:
This work is very nicely carried out and described. It is also impressive to see how the investigators mined data to back up their observations that DNA demethylated regions are enriched for young retrotransposons (LINE-1 elements), which are targets of DNMT3C. One experiment that would have been important to see, potentially underscoring the hypothesis that activation of their repetitive elements in the germline may be associated with diminished germ cells, would be to assay young LINE-1 transcription late in gestation. Moreover, given the surprising observation that there are more methylation defects in sperm from F1 vs F2 mice, and the hypothesis that more adversely affected germ cells are lost in F2 mice, it seems that a comparison of transcription in prospermatogonia in F1 and F2 null mice.

Response: Please see our response above. We agree that these experiments are important. We have modified the text (Discussion, lines 566-574) to indicate what would be required to test our proposed mechanism underlying the worsening of the testicular phenotype in the F2 generation.

Specific (largely minor) comments:
Abstract: “late prenatal germ cell development”. The term prenatal is used a lot in the manuscript but in the abstract, it seems more difficult for this reader to process. How about late fetal or embryonic? This is a bit more intuitive for a developmental biologist.

Response: We agree. We have reviewed our use of the term ‘prenatal’ throughout and have replaced it with the term ‘embryonic’.

Introduction, lines 68-80. In this paragraph, the authors discuss the DNMT enzymatic domains, including the PWWP and ADD domains. Although it is stated what histones these domains interact with, no information is given as to why the interaction is significant. Perhaps an extra sentence

© 2021. Published by The Company of Biologists under the terms of the Creative Commons Attribution License (https://creativecommons.org/licenses/by/4.0/).

would be informative.

Response: As suggested, we have added text to better explain significance (lines 75-79).

Figure 1b: the testis weight and sperm count are clearly lower in F1 null males but not statistically significant. This is because of the small number of animals assayed (3,4). Is it possible to increase number slightly?

Response: This finding in the F1 males confirmed our earlier studies where we had examined reproductive parameters in larger numbers of C57BL/6 F1 males (Chan et al., 2010). In the 2010 study, we reported significant decreases in testes weights and sperm counts in adult mice aged 3.5 and 10 months. Here, our interest was in determining if C57BL/6 strain *Mthfr*$^{-/-}$ F1 males could produce F2 *Mthfr*$^{-/-}$ pups. Practically, over a year of breeding, very few F1 *Mthfr*$^{-/-}$ males (fathers) produced F2 *Mthfr*$^{-/-}$ sons in our colony at McGill University. It is possible that only those F1 males with the least severe testicular defects were able to produce F2 *Mthfr*$^{-/-}$ males. In the current study, since we had not examined F2 males in previous studies, to validate the results in the F2 males, we used results from a second colony (University of Ottawa); these mice were derived from mice from our colony at McGill University.

With respect to the production of F2 null males, Mthfr +/- females were used. How are these animals derived (heterozygous matings)? Is it known if these females have any defects in DNA methylation in their germline?

Response: *Mthfr*$^{+/-}$ females were produced from heterozygous parents. Both *Mthfr*$^{+/-}$ and *Mthfr*$^{-/-}$ females are able to produce pups with no evidence of alterations in fertility in our colonies. This information has been added to the Methods (lines 589-591). To our knowledge, no one has examined the germ cells of the females to determine if they have defects in methylation.

Reviewer 2 Advance Summary and Potential Significance to Field:
In this interesting manuscript the authors provide evidence that paternal deficiency (with or without maternal deficiency) of methylenetetrahydrofolate reductase (MTHFR) can lead to male reproductive abnormalities across two generations. The focus of the work reported is not so much on the reproductive abnormalities as it is on genomic analysis of epimutations (hypomethylation), postulated to be a transmission mechanism of the reproductive defects. The authors investigated wild-type parameters of the DNA-methylation (DNAme) sites sensitive to absence of MTHFR. Both generations of deficient males show considerable loss of sperm DNAme in overlapping regions that are enriched in young retrotransposons. Importantly, these sites appear to be methylated at the time of expression of MTHFR in prenatal male germ cells. Although the findings are intriguing, the authors did not conduct experiments to tie genomic DNAme depletion to the reproductive phenotype and thus the results lack mechanistic significance.

Response: As outlined in the preamble above, we agree that experiments to determine the mechanism underlying our findings are important. We think it is worth reporting our findings earlier rather than later as it may allow other investigators to start looking for similar findings (i.e. inherited hypomethylation of young retrotransposons) to explain epigenetic inheritance in their models.

Reviewer 2 Comments for the Author:
This manuscript is well written and a pleasure to read. The organization is a logical progression through the work. The figures are well organized and laid out, easily interpretable without resorting to the legends, which are nonetheless clear. The findings will be of interest to the community of developmental biologists.

For the most part, the conclusions are supported by the data. One area where this deserves more careful attention is in the last section of Results, with respect to Fig. 6A. Evaluation of these three figure panels by “eyeball statistics” suggests that all three proteins, NSD1,

© 2021. Published by The Company of Biologists under the terms of the Creative Commons Attribution License (https://creativecommons.org/licenses/by/4.0/).

DNMT3L and DNMT3C, play a role in methylation of the MTHFR-sensitive sites, with greatest effect exerted by DNMT3L. Elaboration on the conclusion that DNA methylation at these sites depends on DNMT3L and DNMT3C but not NSD1 would be valuable, as would justification for follow-up on Dnmt3c KO data rather than data from Dnmt3l KOs, where the effect was seemingly the greatest.

Response: The reviewer is correct that the greatest effect on DNA methylation is exerted by DNMT3L. Indeed, DNMT3L is broadly required for DNA methylation during male germline development, including of young TEs (Barau et al , Science, 2016, Figure 3D). This is clearly apparent in Fig. 6A, where the vast majority of the 50,000 random 1 kb bins presented show >75% DNA methylation in WT but between 75% and 0% DNA methylation in the Dnmt3l KO. Therefore, not surprisingly, the same is true for the F1 hypo DMRs, with a strong impact on their DNA methylation levels. The effect of NSD1 is much more modest on the F1 hypo DMRs than it is on the 50,000 random 1 kb bins, indicating that NSD1 is not involved in de novo DNA methylation of the former, consistent with our recent study showing that young TEs are not regulated by NSD1 (Shirane et al., Nature Genetics, 2020). Critically, unlike DNMT3L, DNMT3C has minimal impact on global DNA methylation levels, as can clearly be seen in Figure 6A, third panel. Nevertheless, despite the relatively low impact on DNA methylation overall of the DNMT3C mutant, there is a clear enrichment of DMR regions that overlap with young LINE-1 elements that show increased expression of more than 5-fold in *Dnmt3c* KO compared to *Dnmt3c+/-* (DNMT3C-sensitive) testes (Barau et al , Science, 2016), as shown in Figure 6B. In other words, while DNMT3L is important for DNA methylation of these regions, its role in DNA methylation is not restricted to these regions, while DNMT3C is specifically targeted to young potentially active TEs and was therefore of greater interest. We have edited the Results section and Discussion to emphasize these points.

There is one overriding concern about the focus of this manuscript. In spite of the title of the manuscript, most of the emphasis is on the genomic distribution of DNAme marks (potential epimutations) and how they change in absence of MTHFR. Relatively little attention is given to the phenotype (less than 1 figure of a total of 6 figures). Thus, interesting though they may be, the genomic findings have not been tied mechanistically to the reproductive phenotype except by speculation. Moreover, if the epimutations are responsible for the phenotype, it is puzzling that they are fewer DMTs in F2 sperm than in F1 sperm. As the authors point out, this could be explained by loss of affected germ cells early in their development, but this could be bolstered more extensive analysis of germ-cell death. In summary, while the body of the genomic analysis is impressive, the impact of the genomic findings would be greatly enhanced if they could be better tied to the reproductive defects. For example, determination if LINE 1 elements are or are not mis-expressed in mutant germ cells would inform if the simplest inferred relationship is valid or if the mechanisms are more complex.

Response:
It is likely that detailed developmental testicular histology studies of the F1 fathers and their F2 sons will be required to complement the mechanistic molecular studies we propose above.
We have previously examined the developmental phenotype of F1 generation *Mthfr*$^{-/-}$ mice (Kelly et al., 2005; Chan et al., 2010). The previous studies included detailed examination of germ cell numbers, proliferation and apoptosis as well as adult spermatogenesis. As our previous results may help better connect the testicular phenotype and the proposed timing of the molecular events (at least in the F1 generation), we have added a paragraph to the Discussion (lines 477-502).

As discussed in the General Comments above, in follow-up mechanistic studies, it will be important to examine whether retrotransposons are mis-expressed in mutant germ cells. To help explain the worsening phenotype in F2 versus F1 males, expression of LINE 1 in mutant germ cells would need to be done in both F1 and F2 late fetal and early postnatal testes before germ cells are lost. We would need to perform further breeding experiments to carry out these studies. Such studies are only likely to help determine the mechanism underlying the worsening of the phenotype in the F2 versus the F1 in the context of the full series of experiments described above.

© 2021. Published by The Company of Biologists under the terms of the Creative Commons Attribution License (https://creativecommons.org/licenses/by/4.0/).

Reviewer 3 Advance Summary and Potential Significance to Field:
In the manuscript “Paternal MTHFR deficiency leads to reproductive decline across generations in association with hypomethylation of young retrotransposons”, Karahan et al show that the loss of 5, 10-Methylenetetrahydrofolate reductase (MTHFR) leads to widespread hypomethylation of the sperm genome in F1 and F2 generations. Intriguingly, however, reproductive defect is exacerbated in the F2 mice, while the F1 mice are fertile. Based on these results, the authors propose that decrease of DNA methylation in MTHFR-/- mice causes reproductive decline across generations.

Reviewer 3 Comments for the Author:
The comments for the authors are given below.

1. The authors show reproductive defects specifically in the F2 generation, which is slightly different from reproductive decline taking place across successive generations. As such, it might be better to modify the title so that it represents the results better.

Response:
We identified a significant increase in abnormal tubules in the testes of the F1 males (Fig. 1C). Consistent with an impact on the testes of the F1 males were non-significant decreases in testes weights and sperm counts (Fig. 1B). Testes weights, sperm counts and testicular histology were more markedly affected in the F2 sons. To more accurately represent the results, we suggest changing the title to indicate we are only looking at two generations as follows: “Paternal MTHFR deficiency leads to hypomethylation of young retrotransposons and reproductive decline across two successive generations”

2. The F2 phenotype shown here could also indicate primordial germ cells defects during gestation. Indeed, the authors (Niles et al, PLoS one, 2011) previously reported a similar finding. In the present manuscript, the authors also note that “Based on high levels of expression of MTHFR in prospermatogonia (PSG), MTHFR deficiency is expected to affect F1 generation (1st hit) primordial germ cells (PGCs) when DNA methylation patterns are established” (lines 683-686). Potential aberrations in the PGC methylome should be analyzed for better interpretation of the results.

Response:
As outlined in the General Comments to reviewers section above, to get at underlying mechanisms, we agree that the DNA methylome, in addition to the histone methylome, and the transcriptome would need to be examined in follow-up studies.

3. The reproductive defect discussed in this paper is mostly based on sperm count and testis weight. A more detailed analysis should be required (for example, see Dong et al, Nat Commun, 2019; PMID: 31624244). At which stage(s) of spermatogenesis/spermiogenesis do these defects occur?

Response: We have previously reported detailed studies of the development and basis of the testicular phenotype in F1 *Mthfr*$^{-/-}$ mice with both infertility and subfertility. As also requested by Reviewer 2 (see response above), we have added more description of the previous findings to the text (lines 477-502).

4. The authors propose that aberrant de-repression of retrotransposable elements caused by hypomethylation of the genome, leads to reproductive defects in F2 mice. This claim should be backed up with RNA-seq data showing the actual de-repression of the repeats. Measuring the level of piRNAs (see Dong et al, Nat Commun, 2019) may also be performed.

Response: Please see our General Comments to reviewers response above. As there are reproductive defects in the F1 testis (this study and our previous study Chan et al., 2010) and the F2 testis, it is likely that aberrant de-repression of retrotransposons will be found in male germ cells of the F1 and F2. We propose that the worsening of the phenotype in the F2 is a likely combination of factors that would require the studies mentioned in our General Response and outlined on lines 566-574 of the manuscript.

© 2021. Published by The Company of Biologists under the terms of the Creative Commons Attribution License (https://creativecommons.org/licenses/by/4.0/).

Taken together, the results shown in this paper are certainly interesting. However, the mechanistic explanation of why F2 mice show enhanced reproductive defects needs to be more rigorously explored.

Response: We agree, as noted above, but feel that the required experiments cannot be completed within a reasonable time of revision. We have explicitly stated this limitation at the end of our Discussion (lines 566-574).

Second decision letter

MS ID#: DEVELOP/2021/199492

MS TITLE: Paternal MTHFR deficiency leads to hypomethylation of young retrotransposons and reproductive decline across two successive generations

AUTHORS: Gurbet Karahan, Donovan Chan, Kenjiro Shirane, Taylor McClatchie, Sanne Janssen, Jay M. Baltz, Matthew Lorincz, and Jacquetta Trasler
ARTICLE TYPE: Research Article

I am happy to tell you that your manuscript has been accepted for publication in Development, pending our standard ethics checks.

Reviewer 1

Advance summary and potential significance to field

The results reveal more biology with respect to reprogramming in the male germline.

Comments for the author

While I would have like to see RNAseq as suggested in my review as well as the other two, I can understand the authors' argument against the addition of such data. Thus, I am satisfied with the revision.

Reviewer 2

Advance summary and potential significance to field

As stated in previous review, this paper gives intriguing insight into across-generation effects of paternal MTHFR.

Comments for the author

The rebuttal and revised manuscript adequately address my concerns expressed in the previous review.

While it is disappointing that mechanisms cannot not more readily addressed, I agree that it is important to get these results out. The end of the last Discussion paragraph in the revision sounds quite apologetic; it probably wouldn't take much work to word it more positively with respect to goals, rather than the list of necessary tasks and conditions.

There is an interesting comment hidden in the authors' response: that the subfertility phenotype is lost on a mixed background - do the authors want to give more attention to this in the manuscript?

© 2021. Published by The Company of Biologists under the terms of the Creative Commons Attribution License (https://creativecommons.org/licenses/by/4.0/).

For instance, this observation could suggest the existence of modifier genes, further evidence of the complexity of the phenotype.

The new title is an improvement over the previous one, highlighting the major focus.

Reviewer 3

Advance summary and potential significance to field

The F2 phenotype observed by paternal MTHFR deficiency is certainly interesting, and the model that deregulation of retrotransposons due to DNA methylation defects could lead to such phenotype also makes sense.

Comments for the author

To sufficiently consolidate this model the authors still need to provide some mechanistic explanation to show how DNA methylation defects are linked with reproductive defects.
As suggested in the first round of revision, evidence that repetitive elements are derepressed in the MTHFR deficient sperm (RNA-seq), should be a critical piece of experiment to support the authors' hypothesis.
If the mechanistic explanations are sufficiently given, this paper should definitely be worth publishing.

© 2021. Published by The Company of Biologists under the terms of the Creative Commons Attribution License (https://creativecommons.org/licenses/by/4.0/).
